# Mesencephalic Astrocyte‐Derived Neurotrophic Factor Binds BAX to Preserve Mitochondrial Homeostasis and Energy Metabolism for Relieving Myocardial Hypertrophy

**DOI:** 10.1002/advs.202502835

**Published:** 2025-07-30

**Authors:** Dong Wang, Xinru Zhang, Baolong Wang, Haipeng Li, Dongshuo Xu, Yun Yang, Jialu Zhang, Wenbing Wang, Ren Zhang, Xinyu Wang, Yunfeng Cai, Shiyu Cao, Chao Hou, Changhui Wang

**Affiliations:** ^1^ School of Basic Medical Sciences Anhui Medical University Hefei Anhui 230032 China; ^2^ Department of Cardiology The First Affiliated Hospital of Anhui Medical University Hefei Anhui 230022 China; ^3^ School of Pharmacy Anhui Medical University Hefei Anhui 230032 China

**Keywords:** Bcl2‐associated X protein, cytochrome c, mesencephalic astrocyte‐derived neurotrophic factor, mitochondria, myocardial hypertrophy

## Abstract

Myocardial hypertrophy (MH) is a heart disease accompanied by mitochondrial energy disorder and oxidative stress for cardiomyocyte apoptosis. Mesencephalic astrocyte‐derived neurotrophic factor (MANF), with anti‐inflammation and cytoprotection, is found to be negatively correlated with atrial apoptosis and fibrillation. Here, the effect and mechanism of MANF on MH are studied. Myocardial cell‐specific MANF knockout (MKO) mice are constructed to establish transverse aortic constriction (TAC) or angiotensin II (Ang II)‐induced MH model. MANF is found to be upregulated by MH and protects cardiomyocytes against TAC or Ang II‐induced MH. Mechanistically, through single‐cell RNA sequencing and metabolomics analysis, MANF in cardiomyocytes is closely involved in glycolysis‐oxidative phosphorylation balance and mitochondrial homeostasis. Furthermore, MANF interacts with pro‐apoptotic BAX to inhibit BAX mitochondrial translocation, subsequently decreasing mitochondrial damage, cytochrome c release, and cardiomyocyte death. These results indicate a promising clinical value of MANF for MH treatment, and also preliminarily define MANF's role in mitochondrial energy production and mitochondria‐associated apoptosis pathway.

## Graphical abstract

MANF in cardiomyocytes interacts with BAX to impede BAX mitochondrial translocation and cytochrome c release, further stabilizing mitochondrial structure and energy supply to relieve myocardial apoptosis and hypertrophy, which indicates MANF as a novel cardioprotective factor to have a promising clinical application for myocardial hypertrophy.

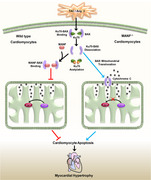

## 1 Introduction

The pathological myocardial hypertrophy (MH) is a cardiac adaptive response to pathological stimuli under overloaded conditions, which further causes cardiac dysfunction and failure.^[^ ^1^, ^2^ ^]^ Angiotensin II (Ang II) overstimulation and transverse aortic constriction (TAC) are two common pathological factors for MH.^[^ ^3^ ^]^ Ang II promotes vascular smooth muscle contraction to induce hypertension and cardiac overload.^[^ ^4^ ^]^ It binds to angiotensin type 1 receptor for peroxides overproduction via nicotinamide adenine dinucleotide phosphate oxidase to be involved in MH.^[^ ^5^ ^]^ Also, TAC induces oxidative stress in myocardial tissues for MH occurrence and progression.^[^ ^6^ ^]^ Therefore, Ang II and TAC have often been used to construct MH experimental animal models.^[^ ^6^, ^7^ ^]^

The heart is the most energy‐consuming organ, and energy metabolism occupies a critical role in the normal heart function.^[^ ^8^ ^]^ The impaired cardiac energy metabolism has been reported as an important cause of cardiac dysfunction and myocardial damage.^[^ ^9^, ^10^ ^]^ As the energy supply center, mitochondria are abundant in myocardial cells for ATP production.^[^ ^11^ ^]^ Otherwise, mitochondrial malfunction triggers the attenuated mitochondrial membrane potential, over‐accumulation of reactive oxygen species (ROS), and release of mitochondrial cytochrome c (Cyt‐c) into the cytoplasm for myocardial apoptosis.^[^ ^12^ ^]^ It has been reported that mitochondrial perturbation is a direct contributing factor for MH via ROS accumulation, inflammatory response, and others.^[^ ^13^, ^14^ ^]^ Maintenance of the intact mitochondrial structure and function is regarded as a precondition for cardiocytes' survival and MH prevention.

Mesencephalic astrocyte‐derived neurotrophic factor (MANF) is a small molecular protein for neuroprotection,^[^ ^15^, ^16^ ^]^ which is involved in unfolded protein response and endoplasmic reticulum stress.^[^ ^17^ ^]^ Moreover, MANF acts as an immunoregulation factor to regulate macrophage polarization for inflammation inhibition in the liver, kidney, and lung.^[^ ^18^, ^19^, ^20^, ^21^ ^]^ For the heart, our previous reports have shown that MANF is negatively correlated with atrial apoptosis and fibrillation,^[^ ^22^ ^]^ and mono‐macrophage‐derived MANF relieves bacterial myocarditis via suppression of NF‐κB activation and pro‐inflammatory macrophage differentiation.^[^ ^23^ ^]^ Other researchers have demonstrated an anti‐hypertrophic role of MANF to protect against ischemic heart damage.^[^ ^24^ ^]^ Recently, the effect of MANF on intracellular metabolism has been greatly focused on to reveal its roles in supporting glucose homeostasis, lipid metabolism, and energy balance.^[^ ^25^ ^]^ However, there is still insufficient evidence to disclose the relationship between MANF and mitochondrial stability.

In this study, we further extended our previous research on MANF and cardiac diseases to focus on myocardial cell‐derived MANF's influence and mechanism on MH. Myocardial cell‐specific MANF knockout (MKO) mice were constructed to establish TAC or Ang II‐induced MH mice model for single‐cell RNA sequencing (scRNA‐seq), high‐throughput proteomic screening, and metabolomics analysis. Then, we explored the effect of myocardial cell‐derived MANF on MH development. Also, we preliminarily demonstrated the mechanism of how myocardial apoptosis and mitochondrial homeostasis are regulated by MANF in cardiocytes. These results highlight the potential clinical significance of MANF to MH prevention and treatment, and also expand MANF's role in energy metabolism and mitochondrial dysfunction‐triggered apoptosis.

## 2 Results

### 2.1 Myocardial Hypertrophy Promoted MANF Expression in Heart

**Figure 1:**
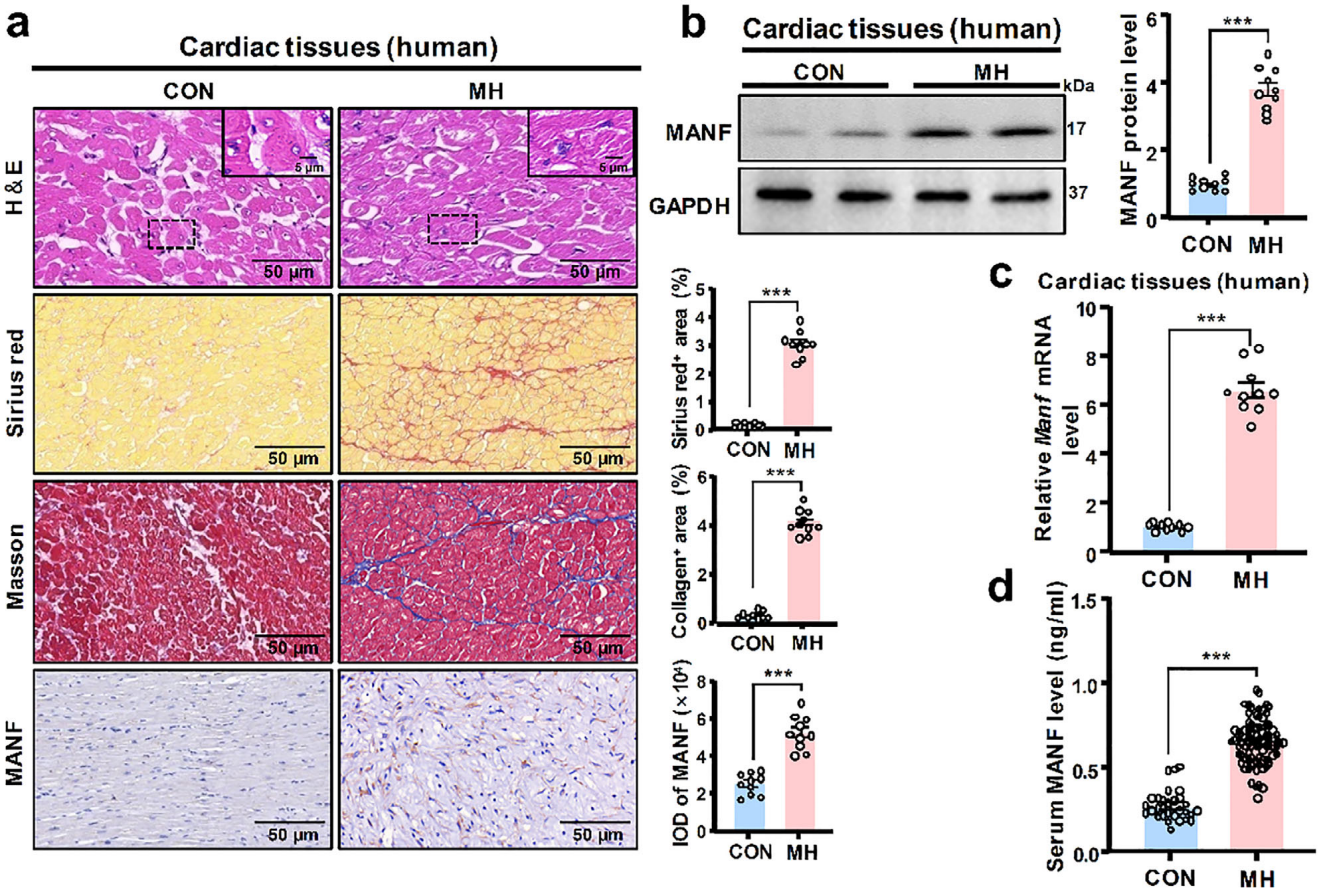
MANF level was greatly increased in cardiac tissues and serum samples of MH patients. Human cardiac tissues of the left ventricle were collected from 10 MH patients (*n* = 10) and 10 healthy donors (*n* = 10), respectively. a) HE, Sirius red, Masson staining, and MANF immunohistochemistry were performed. Sirius red positive area, collagen positive area, and integral optical density of MANF were calculated (*n* = 10). Scale bar: 50 or 5 µm. b) WB and c) qRT‐PCR (*n* = 10) were performed to evaluate MANF protein and mRNA levels. Human serum samples were collected from 80 MH patients (*n* = 80) and 30 healthy volunteers (*n* = 30), respectively. d) ELISA was performed to examine the serum MANF level. Data are expressed as mean ± SD. ^***^ *p* < 0.001. CON: healthy control; MH: myocardial hypertrophy. Data are representative of three independent experiments.

**Figure 2:**
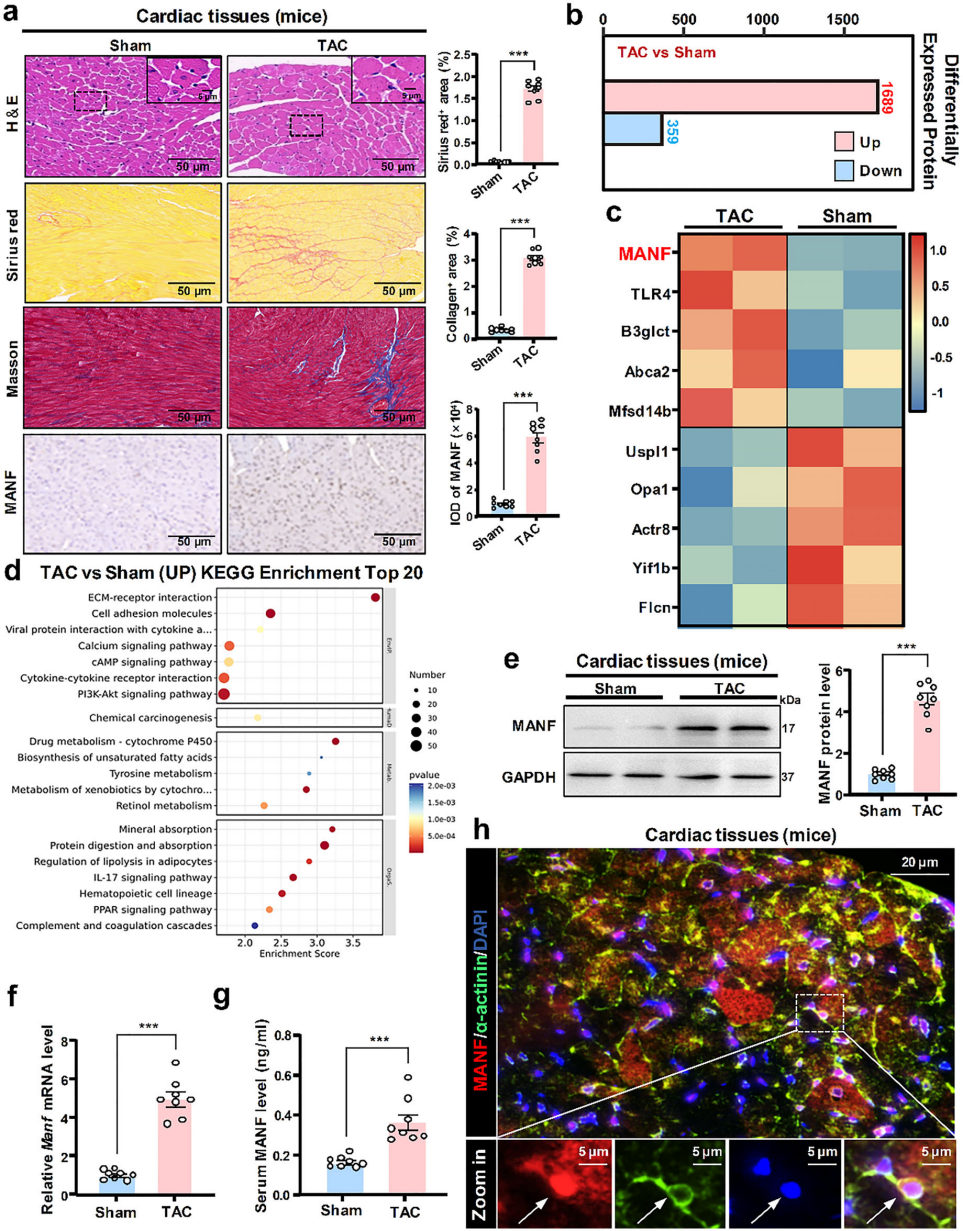
MANF expression was significantly enhanced in cardiac tissues and serum samples of TAC‐induced MH mice. At the fifth week after TAC surgery, cardiac tissues and serum samples were collected from 8 Sham mice (*n* = 8) and 8 TAC mice (*n* = 8), respectively. a) HE, Sirius red, Masson staining, and MANF immunohistochemistry were performed. Sirius red positive area, collagen positive area, and integral optical density of MANF were calculated (*n* = 8). Scale bar: 50 or 5 µm. High‐throughput proteomic screening (*n* = 3) was performed for analysis of differentially expressed genes b, c), and KEGG Enrichment d). e) WB and f) qRT‐PCR (*n* = 8) were performed to evaluate MANF protein and mRNA levels. g) ELISA (*n* = 8) was performed to examine the serum MANF level. h) Immunofluorescent double‐labelled staining of MANF (Red) and α‐actinin (Green) was performed (*n* = 8). DAPI (Blue) was used for nuclei staining. Scale bar: 20 or 5 µm. Data are expressed as mean ± SD. ^***^ *p* < 0.001. Sham: sham operation; TAC: transverse aortic constriction. Data are representative of three independent experiments.

Our previous studies have demonstrated a positive role of MANF in cardiocyte protection, as well as the increased MANF level in atrial fibrillation and bacterial myocarditis.^[^ ^22^, ^23^ ^]^ To further explore the protective effect of MANF on myocardial cells, we focused on the change and function of myocardial cell‐derived MANF in the MH pathological process. Clinically, cardiac tissues and serum samples were collected from MH patients. Results of HE, Sirius red, and Masson staining in **Figure** **1**a showed structural changes and collagen accumulation in cardiac tissues of MH patients. MANF mRNA and protein levels were greatly increased in MH cardiac tissues compared with healthy controls (Figure 1a–c). Besides, the serum MANF level was elevated when MH occurred (Figure 1d). To simulate MH pathogenesis in mice, we constructed MH mice model via TAC or Ang II treatment. **Figures** **2**a and S1a (Supporting Information) showed MH‐featured cardiac structural changes and collagen accumulation after TAC or Ang II treatment. Through high‐throughput proteomic screening, we found that MANF had a ≈1.5‐fold increase in TAC‐induced MH cardiac tissues (Figure 2b–d), which was confirmed by results of WB, qRT‐PCR, and ELISA (Figure 2e–g). Similarly, the raised MANF level in cardiac tissues and serum was found after Ang II treatment (Figure S1a–d, Supporting Information). Both TAC and Ang II‐induced cardiac MANF increases showed to be time‐dependent (Figure S1e,f, Supporting Information). α‐actinin has often been used as a marker of cardiomyocytes in published research.^[^ ^26^, ^27^, ^28^, ^29^ ^]^ As shown in Figure 2h and Figure S1g,h (Supporting Information), the immunofluorescence co‐localization of α‐actinin and MANF indicated that myocardial cells were the main sources of MANF in the heart. These results suggest that MH can significantly promote cardiac MANF expression for both humans and mice, revealing a potential key role of MANF in MH occurrence and development.

### 2.2 Myocardial Cell‐Specific MANF Knockout Aggravated Myocardial Hypertrophy and Cardiac Dysfunction

**Figure 3:**
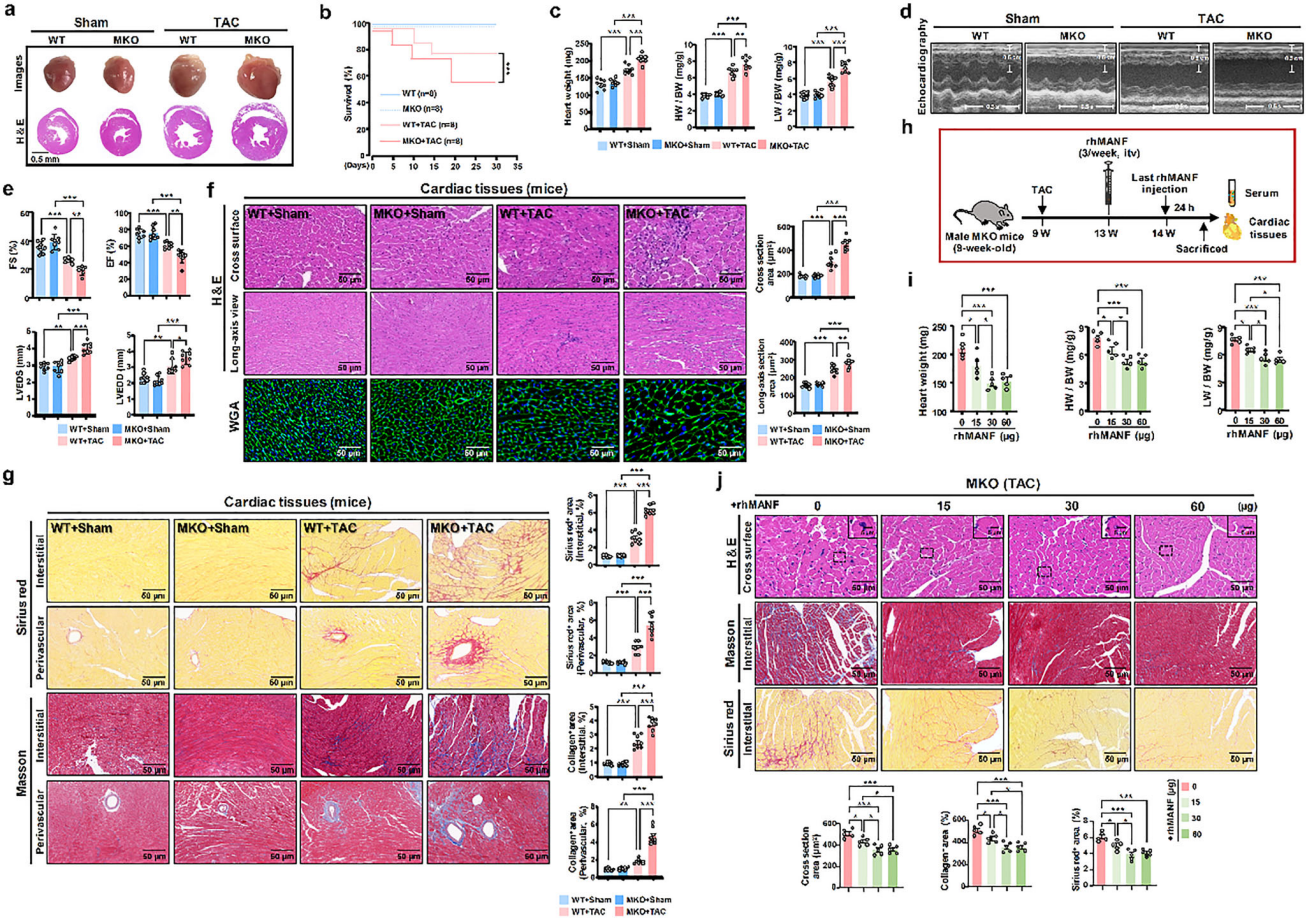
Myocardial cell‐derived MANF alleviated TAC‐induced myocardial hypertrophy and cardiac dysfunction. WT and MKO mice were used to construct TAC‐induced MH model for Sham‐WT (*n* = 8), Sham‐MKO (*n* = 8), TAC‐WT (*n* = 8), and TAC‐MKO (*n* = 8) groups. At the fifth week after TAC surgery, cardiac tissues were collected. a) Heart images were collected, and HE staining of cardiac tissues was performed. Scale bar: 0.5 mm. b) Mice's survival rate was recorded once every five days. c) Heart weight (HW), lung weight (LW), and body weight (BW) were calculated, respectively for ratios of HW/BW and LW/BW (*n* = 8). d,e) Cardiac function was monitored by echocardiography (*n* = 8) at the fifth week after TAC surgery. Cardiac tissues (*n* = 8) were used for HE and WGA staining f), Sirius red and Masson staining g). HE staining included cross‐surface and long‐axis views. Sirius red and Masson staining included interstitial and perivascular views. Cross‐section and long‐axis section areas of HE staining, Sirius red positive area, and collagen positive area were calculated. Scale bar: 50 µm. MKO mice were used to construct TAC‐induced MH model, followed by rhMANF (0, 15, 30, 60 µg) treatment (*n* = 5). h) Diagram of the procedure of TAC‐induced MH mice treated by rhMANF. At the fourth week after TAC surgery, rhMANF was intravenously injected three times per week. i) Heart weight (HW), lung weight (LW), and body weight (BW) were calculated, respectively for ratios of HW/BW and LW/BW (*n* = 5). j) Cardiac tissues (*n* = 5) were used for HE (cross‐surface), Sirius red (interstitial view), and Masson (interstitial view) staining. Scale bar: 50 or 5 µm. Data are expressed as mean ± SD. ^*^ *p* < 0.05, ^**^ *p* < 0.01, ^***^ *p* < 0.001. Sham: sham operation; TAC: transverse aortic constriction; WT: wild type; MKO: myocardial cell‐specific MANF knockout; rhMANF: recombinant human MANF. Data are representative of three independent experiments.

To better examine the effect of myocardial cell‐derived MANF on MH, myocardial cell‐specific MANF knockout mice were established for TAC or Ang II‐induced MH mice models (Figure S2a–d, Supporting Information). After MANF deficiency in myocardial cells, mice's heart volume and weight were partly increased when suffering from TAC or Ang II treatment, along with the descending survival rate (**Figure** **3**a–c; Figure S3a–c, Supporting Information). Considering the close interaction between heart and lung, we also found the higher lung weight in MKO mice with MH (Figure 3c; Figure S3c, Supporting Information). For cardiac function, echocardiography analysis showed the impaired cardiac functional indicators after TAC or Ang II administration, and MKO mice had more severe cardiac dysfunction compared to WT mice, including the weakened fractional shorting (FS)/ejection fraction (EF) and expanded left ventricular end‐diastolic diameter (LVEDD)/left ventricular end‐systolic dimension (LVEDS) (Figure 3d,e; Figure S3d,e, Supporting Information). In the aspect of cardiac tissue structure, HE staining of Figure 3f and Figure S3f (Supporting Information) showed that myocardial cell‐specific MANF knockout was able to cause more aggravated destruction of cardiac tissues induced by TAC or Ang II from both views of cross surface and long axis. As shown in Figure 3g and Figure S3g (Supporting Information), more collagens were accumulated in both interstitial and perivascular areas of MKO cardiac tissues compared with WT mice. Moreover, we performed WGA staining to further verify cardiomyocyte hypertrophy, which showed a significantly increased myocyte diameter and size in MKO mice than that in WT mice when TAC or Ang II was administered (Figure 3f; Figure S3f, Supporting Information). Altogether, these data indicate that myocardial cell‐derived MANF enables partial protection against TAC or Ang II‐induced MH.

### 2.3 Myocardial Hypertrophy in MKO Mice was Relieved by Recombinant Human MANF Treatment

We have previously reported that recombinant human MANF (rhMANF) protein can relieve carbon tetrachloride (CCl_4_)‐induced hepatic fibrosis^[^ ^18^ ^]^ and lipopolysaccharide (LPS)‐triggered myocarditis,^[^ ^23^ ^]^ and rhMANF can be used in mice without any species difference or immune response. Next, we wondered whether rhMANF could reverse the deteriorated cardiomyocyte hypertrophy of TAC or Ang II‐treated MKO mice via exogenous MANF supplement. rhMANF was introduced to treat MKO mice with TAC or Ang II‐induced MH, which was demonstrated in Figure 3h and Figure S4a (Supporting Information). With the increased use of rhMANF from 0 to 60 µg once per mouse, both TAC and Ang II‐induced MH of MKO mice was gradually alleviated, including the reduced heart and lung weight (Figure 3i; Figure S4b, Supporting Information), as well as the relatively mitigated cardiac tissue damage and collagen accumulation (Figure 3j; Figure S4c, Supporting Information). We also found that rhMANF could partly weaken TAC and Ang II‐induced MH in WT mice (Figure S4d, Supporting Information), indicating rhMANF as a promising drug for MH clinical intervention. Therefore, rhMANF is capable of attenuating the serious MH caused by TAC or Ang II.

### 2.4 Myocardial Cell‐Specific MANF Knockout Negatively Affected Energy Metabolic Pathways in Myocardial Cells

**Figure 4:**
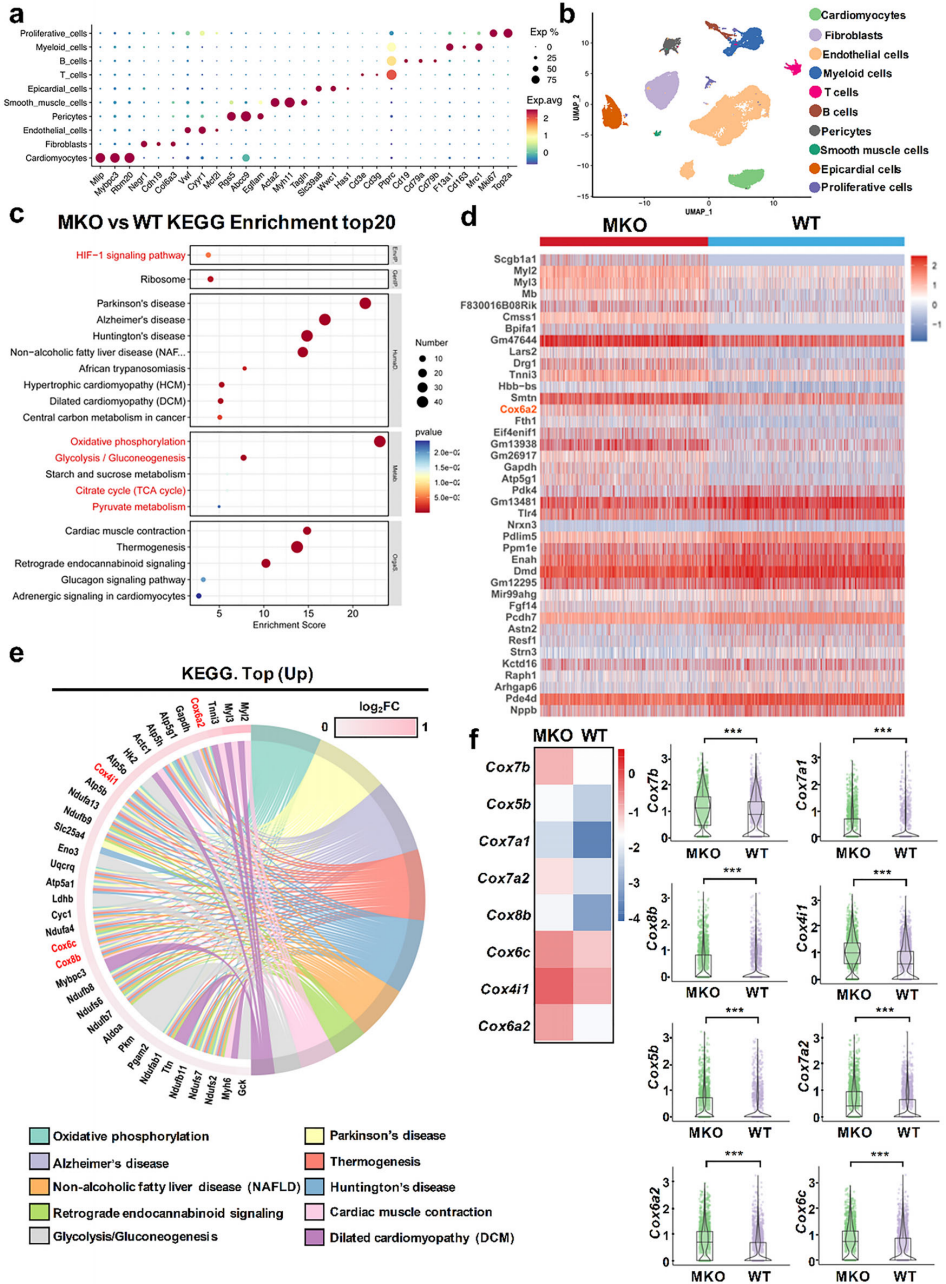
scRNA‐seq analysis for TAC‐induced myocardial hypertrophy in WT and MKO mice. WT and MKO mice were used to construct a TAC‐induced MH model for TAC‐WT (*n* = 6) and TAC‐MKO (*n* = 6) groups, followed by scRNA‐seq analysis. a) Cardiomyocytes identification by Mlip, Mybpc3, and Rbm20 in scRNA‐seq analysis. b) tSNE map to show unsupervised clustering demonstrating 10 distinct cell types. c) Analysis of top 20 enriched pathways. d) Heatmap showing the expression level of DEGs. e) Chord diagram of enrichment analysis to show top 10 pathways of differential genes enrichment. f) Heatmap to show DEGs’ changes of the Cox protein family. The expression level of DEGs and the Cox protein family is evaluated by color change. Color from blue to red means gene products from low to high expression. Data are expressed as mean ± SD. ^***^ *p* < 0.001. WT: wild type; MKO: myocardial cell‐specific MANF knockout.

To disclose the in‐depth mechanism of how myocardial cell‐derived MANF is involved in MH, we used WT and MKO mice with TAC‐induced MH for scRNA‐seq analysis. The procedure of scRNA‐seq sample preparation was demonstrated in Figure S5a, Supporting Information. First, 10 distinct cell populations were identified, including cardiomyocytes (**Figure** **4**a,b; Figure S5b, Supporting Information). Subsequently, differentially expressed genes (DEGs) between WT and MKO cardiomyocytes in TAC‐induced MH were involved in the analysis of heatmap, KEGG pathway enrichment, volcano plot, and others. KEGG enrichment analysis in Figure 4c showed that multiple energy metabolism‐associated pathways of gluconeogenesis, TCA cycle, pyruvate metabolism, especially oxidative phosphorylation, were significantly affected by myocardial cell‐specific MANF knockout; also, Figure S6a (Supporting Information) revealed the consistent changes of ATP biosynthesis and metabolism pathways. After a further analysis, we found that there was a great expression difference of multiple cytochrome c oxidase (Cox) gene family members after MANF deficiency in MH cardiomyocytes, including Cox7b, Cox5b, Cox7a1, Cox7a2, Cox8b, Cox6c, Cox4i1, and Cox6a2 (Figure 4d–f; Figure S6b,c, Supporting Information). Interestingly, all differentially expressed Cox genes showed consistent up‐regulation in MKO cardiomyocytes compared with WT (Figure 4f; Figure S6c, Supporting Information), particularly Cox6a2 with the most remarkable up‐regulation (Figure 4d; Figure S6b, Supporting Information). Moreover, Figure S6d,e (Supporting Information) verified the significantly increased mitochondrial Complex IV level and mRNA transcripts of Cox6a2 and Cox6c in TAC‐induced MKO primary cardiocytes. Otherwise, complex I, II/III, and IV's enzymatic activity showed a significant decline after MANF knockout (Figure S6f, Supporting Information). However, there was no significant change in Cox and other energy metabolism‐related genes between normal WT and MKO primary cardiomyocytes, indicating that MANF failed to affect cardiomyocytes’ energy supply in the basal nonpathological condition (Figure S6g, Supporting Information). These findings indicate that ATP production‐related energy metabolism in cardiomyocytes is regulated by MANF under the MH pathological condition.

### 2.5 Myocardial Cell‐Specific MANF Knockout Triggered the Unbalance of Glycolysis and Mitochondrial Oxidative Phosphorylation

**Figure 5:**
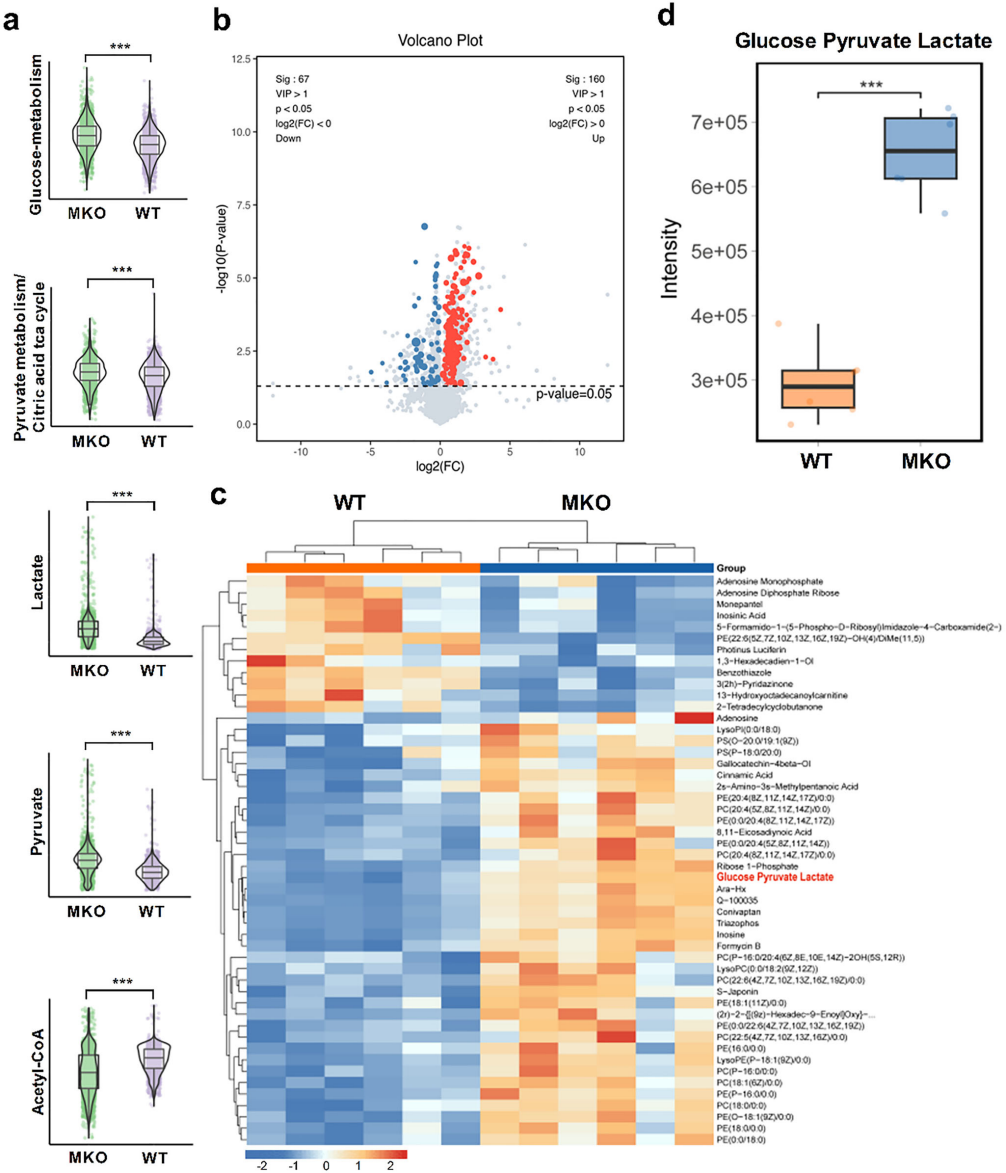
Changes of energy metabolic pathways between WT and MKO mice in TAC‐induced myocardial hypertrophy. WT and MKO mice were used to construct a TAC‐induced MH model for TAC‐WT (*n* = 6) and TAC‐MKO (*n* = 6) groups, followed by scRNA‐seq and metabolomics analysis. a) scRNA‐seq analysis for DEGs’ changes in metabolic pathways and metabolites. b) Volcano plot of metabolomics analysis. Red, blue, and gray dots represent metabolites with significant up‐regulation, significant down‐regulation, and no significant difference, respectively. c) Heatmap of metabolomics analysis. The expression abundance of metabolites is evaluated by color change. Color from blue to red means metabolites from low to high abundance. d) Boxplot of metabolomics analysis to evaluate differential metabolites of glucose, pyruvate, and lactate. Data are expressed as mean ± SD. ^***^ *p* < 0.001. WT: wild type; MKO: myocardial cell‐specific MANF knockout.

ATP biosynthesis and energy supply in cardiomyocytes come from multiple ways of fatty acid oxidation, oxidative phosphorylation, glycolysis, and others.^[^ ^30^ ^]^ Myocardial damage and dysfunction often occur along with the fortified glycolysis, and the degree of glycolysis partly reflects the integrity of myocardial structure and function.^[^ ^9^, ^31^, ^32^ ^]^ Next, a further analysis of scRNA‐seq focused on glucose‐related energy metabolism, which showed that DEGs of myocardial cells could enhance glucose‐pyruvate‐lactate metabolism in MKO mice, accompanied by an increase of lactate and pyruvate metabolites (**Figure** **5**a). Furthermore, we conducted ^13^C‐glucose metabolic flux and metabolomics analysis to verify scRNA‐seq results. Metabolic flux analysis in Figure S7 (Supporting Information) demonstrated that MANF deficiency in cardiocytes promoted glucose metabolism to be more biased to cytoplasmic glycolysis and pyruvate‐lactate pathway under Ang II stimulation. There was no difference in glycolysis metabolites between WT and MKO cardiac tissues under normal basal conditions (Figure S8a,b, Supporting Information). As shown in Figure 5b, a total of 67 down‐regulated and 160 up‐regulated metabolites were found in MKO cardiac tissues compared to WT under TAC‐induced MH pathological conditions. Heatmap and boxplot of metabolomics analysis revealed glycolysis metabolites of glucose, pyruvate, and lactate were greatly increased in MKO cardiac tissues, which was consistent with scRNA‐seq analysis (Figure 5c,d). Differently, acetyl‐CoA, as an important intermediate metabolite of oxidative phosphorylation in mitochondria,^[^ ^33^ ^]^ was found to be significantly down‐regulated in TAC‐induced MH tissues of MKO mice (Figure 5a), indicating the damaged oxidative phosphorylation and deficient mitochondrial function. All scRNA‐seq, metabolic flux, and metabolomics analysis imply that MANF is a critical factor for maintenance of the intact mitochondrial function to ensure the balanced glycolysis‐oxidative phosphorylation and stable energy supply in myocardial cells.

### 2.6 Myocardial Cell‐Specific MANF Knockout Exacerbated Mitochondrial Disorder and Cardiomyocyte Apoptosis in Myocardial Hypertrophy

**Figure 6:**
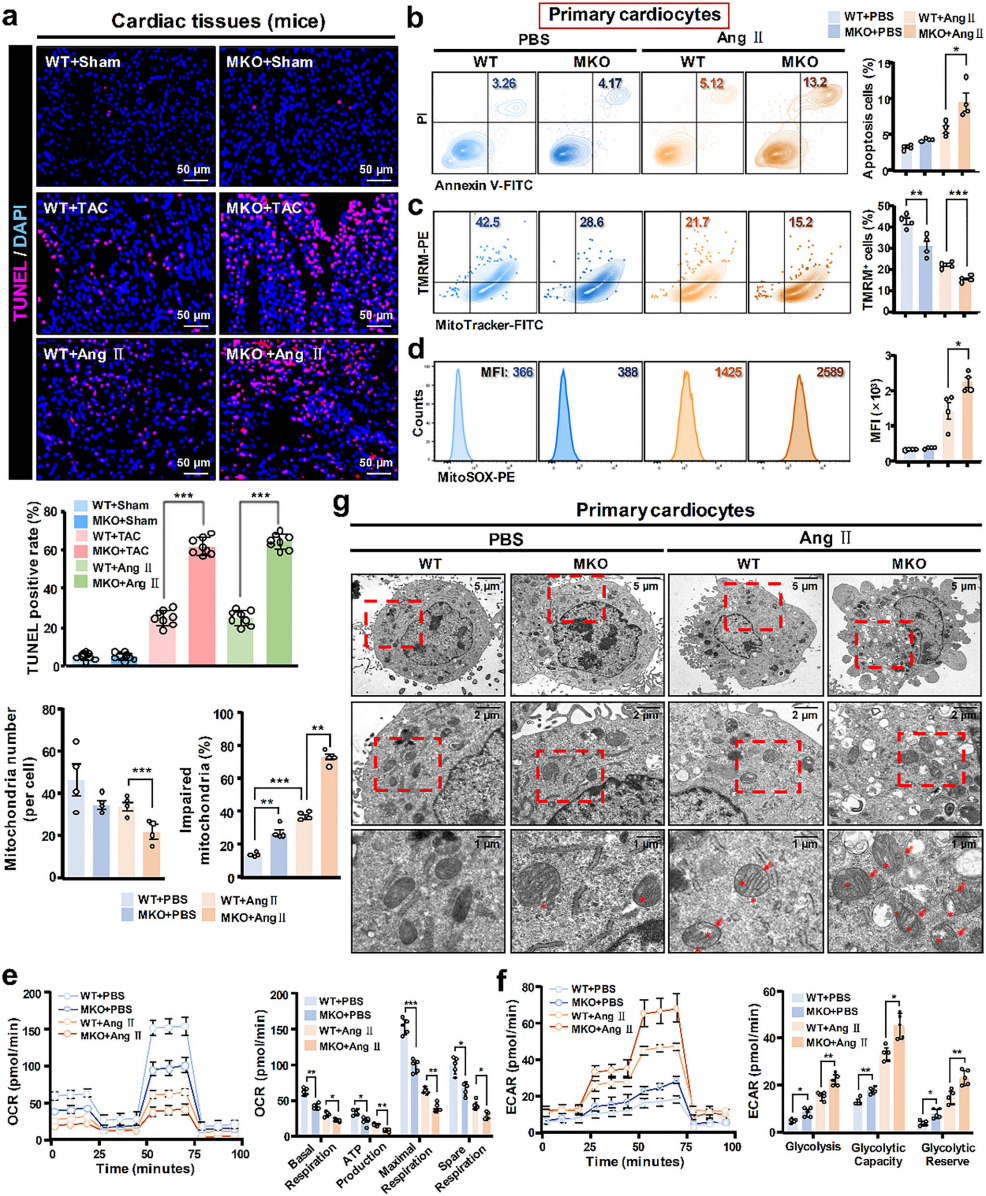
Myocardial cell‐derived MANF maintained mitochondrial stability and function to ensure cardiomyocyte survival. a) TUNEL assay for cardiac tissues of WT and MKO mice with TAC or Ang II‐induced MH model (*n* = 8). Scale bar: 50 µm. The TUNEL positive rate was calculated. Primary cardiocytes (1 × 10^5^ cells/sample) were extracted from WT and MKO mice, followed by Ang II (0.2 µm) treatment for 48 h. Annexin V‐FITC/PI apoptosis detection b), MitoTracker‐FITC/TMRM‐PE mitochondrial membrane potential detection c), and MitoSOX‐PE mitochondrial oxidative stress detection d) were performed. OCR e) and ECAR f) assays were performed to evaluate oxidative phosphorylation and glycolysis, respectively. g) After fixing, centrifugation, dehydration, embedding, and ultrathin section, TEM scanning was performed to evaluate mitochondria damage of mitochondrial swelling, cristae vague and disorder in primary cardiocytes (1 × 10^6^ cells/sample). Scale bar: 5, 2, or 1 µm. The average mitochondria number and the proportion of impaired mitochondria were calculated. Data are expressed as mean ± SD. ^*^ *p* < 0.05, ^**^ *p* < 0.01, ^***^ *p* < 0.001. Sham: sham operation; TAC: transverse aortic constriction; Ang II: angiotensin II; PBS: phosphate buffer saline; WT: wild type; MKO: myocardial cell‐specific MANF knockout. Data are representative of three independent experiments.

Mitochondrial instability triggers cardiomyocyte apoptosis that further positively correlates with MH.^[^ ^34^, ^35^, ^36^, ^37^ ^]^ To further figure out what effects are caused by myocardial cell‐specific MANF knockout on mitochondrial homeostasis and cardiomyocyte apoptosis, we performed TUNEL assay, Annexin V‐FITC/PI FCM, mitochondrial membrane potential, ROS, and Caspase‐3/9 detections by cardiac tissues and primary cardiomyocytes. Results of the TUNEL assay showed that myocardial cell‐specific MANF knockout significantly aggravated TAC or Ang II‐triggered myocardial apoptosis (**Figure** **6**a); similarly, primary MKO cardiocytes had greater Annexin V^+^PI^+^ apoptotic staining and higher Cleaved caspase‐3/9 levels after Ang II treatment (Figure 6b; Figure S9a, Supporting Information). As shown in Figure 6c,d, cardiomyocyte MANF was also positively associated with maintenance of mitochondrial membrane potential and mitochondrial ROS elimination. Furthermore, results from OCR and ECAR assays showed that cardiomyocyte MANF was greatly involved in oxidative phosphorylation and glycolysis. Under Ang II treatment, primary MKO cardiocytes showed a lower oxygen consumption rate, representing the restrained mitochondrial ATP production, basal, maximal, and spare respiration, along with a higher extracellular acidification rate covering the fortified glycolysis, glycolytic capacity and reserve (Figure 6e,f). After rhMANF rescue, TAC or Ang II‐caused cardiac apoptosis in MKO mice was partly reversed (Figure S10a, Supporting Information). Consistently, the aggravated apoptosis, mitochondrial membrane potential maladjustment, and ROS accumulation of primary MKO cardiocytes under Ang II stimulation were also significantly rescued by rhMANF (Figure S10b–d, Supporting Information), along with the partly restored oxygen consumption and inhibited extracellular acidification (Figure S10e,f, Supporting Information). Structurally, TEM scanning images in Figure 6g showed Ang II‐induced structural abnormalities and damages of mitochondria in primary WT and MKO cardiocytes, including mitochondria decrease, mitochondrial swelling, cristae vague and disorder. Myocardial cell‐specific MANF knockout greatly decreased mitochondrial number and made mitochondria damage more deteriorated in Ang II‐treated primary cardiocytes. Therefore, MANF is indispensable for mitochondrion structural integrity and functional stability in myocardial cells to promote cardiomyocyte survival. Additionally, mitophagy has been reported to be greatly beneficial for cardioprotection and MH relief,^[^ ^38^, ^39^ ^]^ especially PINK1/Parkin‐mediated mitophagy^[^ ^40^ ^]^ that is regulated by MANF‐PRKN (parkin RBR E3 ubiquitin protein ligase) interaction.^[^ ^41^ ^]^ In primary WT cardiocytes, mitochondrial PINK1 and Parkin were greatly increased by Ang II treatment, along with a reduced cytoplasmic p62 (mitophagy‐related protein) level. After MANF deficiency, the increased PINK1/Parkin and decreased p62 were partly reversed (Figure S9b, Supporting Information). These data indicate that mitophagy is another potential channel for MANF's positive role in apoptosis resistance and myocardial protection in MH progress.

### 2.7 MANF Interacted with BAX to Impede Mitochondrial Membrane Translocation and Cytochrome C Release

**Figure 7:**
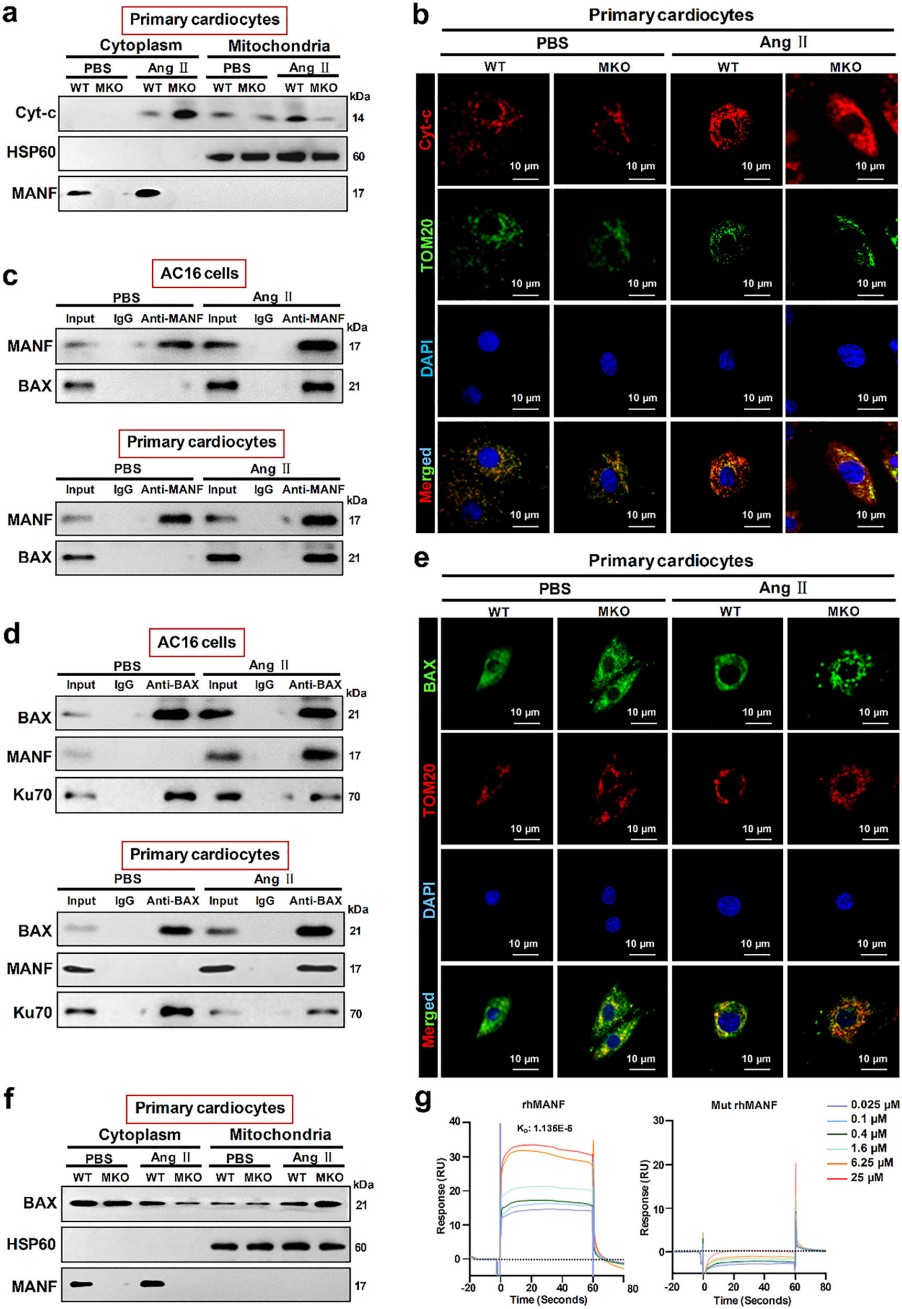
The binding of MANF and BAX to attenuate Ang II‐induced BAX mitochondrial translocation and Cyt‐c release. Primary cardiocytes (1 × 10^6^ cells/sample) were extracted from WT and MKO mice, followed by Ang II (0.2 µm) treatment for 48 h. a) Cell fractionation was performed to collect cytoplasm and mitochondrial proteins, followed by WB for Cyt‐c, HSP60, and MANF. b) Immunofluorescent double labelled staining of Cyt‐c (Red) and TOM20 (Green) was performed with nuclei DAPI (Blue) staining. Scale bar: 10 µm. AC16 and primary WT cardiocytes (1 × 10^6^ cells/sample) were treated by Ang II (0.2 µm) for 48 h, followed by Co‐IP using MANF antibody for BAX detection c), BAX antibody for MANF and Ku70 detection d). For Ang II‐treated primary WT and MKO cardiocytes (1 × 10^6^ cells/sample), e) Immunofluorescent double labelled staining of BAX (Green) and TOM20 (Red) was performed with nuclei DAPI (Blue) staining. Scale bar: 10 µm. f) Cell fractionation was performed to collect cytoplasm and mitochondrial proteins, followed by WB for BAX, HSP60 and MANF. g) Surface plasmon resonance analysis for BAX binding with rhMANF or Mut rhMANF. PBS: phosphate buffer saline; Ang II: angiotensin II; WT: wild type; MKO: myocardial cell‐specific MANF knockout; rhMANF: recombinant human MANF; Mut rhMANF: Mutated recombinant human MANF. Data are representative of three independent experiments.

There is a sort of cell death mediated by Bcl2‐associated X protein (BAX) mitochondrial membrane translocation and cytochrome c release from mitochondria to cytoplasm.^[^ ^42^ ^]^ Ku70 is able to interact with BAX for BAX cytoplasmic retention, and Ku70 acetylation promotes Ku70‐BAX dissociation and BAX mitochondrial localization.^[^ ^43^ ^]^ BAX binding function of Ku70 greatly depend on its C‐terminal SAP domain, likewise MANF's C‐terminal domain shares a high homology and sequence similarity with Ku70's C‐terminal domain.^[^ ^44^ ^]^ We accordingly speculate that MANF may have a similar role as Ku70 for BAX binding. First, we found that Ang II promoted Cyt‐c translocation from mitochondria to cytoplasm in primary cardiocytes, and MANF deficiency triggered more mitochondrial Cyt‐c to enter cytoplasm (**Figure** **7**a), which was supported by immunofluorescence results in Figure 7b showing the greater Cyt‐c diffused distribution in Ang II‐treated MKO cardiocytes compared with WT cardiocytes. Second, using primary cardiocytes and the human myocardial AC16 cell line, intracellular MANF could interact with BAX after Ang II treatment (Figure 7c,d); besides, Ang II induced a decrease of Ku70‐BAX binding (Figure 7d), which contributed to BAX mitochondrial translocation and cardiomyocyte apoptosis.^[^ ^45^ ^]^ In Figure 7e, we observed that Ang II‐triggered BAX dotted colocalization with mitochondrial marker TOM20 was significantly enhanced by myocardial cell‐specific MANF knockout. Also, the results of Figure 7f further verified the increased BAX mitochondrial translocation from cytoplasm in MKO cardiocytes after Ang II stimulation. Additionally, we constructed MANF C‐terminal mutant (Mut C‐MANF) with the conservative VKELKK motif changed to be IKEIKK (residues 113–118). Through Surface Plasmon Resonance (SPR) experiments, MANF‐BAX binding was verified, but recombinant human Mut C‐MANF protein failed to interact with BAX, suggesting a critical role of MANF's C‐terminal domain for BAX binding, especially VKELKK motif (Figure 7g). We also found Mut C‐MANF failed to rescue MKO cardiocytes from TAC or Ang II‐induced apoptosis (Figure S10a, Supporting Information). To further explore the effects of BAX and glycolysis on MH progress, BAX Inhibitor Peptide V5 (BIP V5) and 2‐Deoxyglucose (2DG) were used to treat TAC or Ang II‐induced MH in MKO mice. BIP V5 significantly ameliorated MH of MKO mice, comparatively MH process was partly boosted by 2DG treatment (Figure S11, Supporting Information), which was consistent with the previously verified cardiotoxicity of 2DG as a glycolysis inhibitor.^[^ ^46^ ^]^ These data indicate that cardiomyocyte MANF is a substitute protein for BAX binding to impede BAX mitochondrial translocation and mitigate Cyt‐c mitochondrial release under Ang II‐induced MH pathological status.

## 3 Discussion

Energy supply maladjustment is a common characteristic and inducement for multiple cardiac diseases like arrhythmia and myocardial hypertrophy.^[^ ^47^ ^]^ For cardiomyocytes, ≈90% of total ATP demand is provided by mitochondrial oxidative phosphorylation, which makes mitochondrial malfunction a start for ATP deficit and heart failure.^[^ ^14^ ^]^ External pathogenic factors often initially cause mitochondrial turbulence and oxidative phosphorylation disorder for obstructing of ATP production, further resulting in a metabolic status of strong ATP demand and excessive ROS accumulation that triggers oxidative stress with more serious mitochondrial damage and lower ATP production efficiency.^[^ ^48^ ^]^ Eventually, a vicious circle from mitochondrial dysfunction, ATP insufficiency, to ROS over‐accumulation is formed to deteriorate cardiac diseases.^[^ ^14^ ^]^ Thus, MH is often seen as a sort of mitochondrial disease.^[^ ^49^ ^]^ Mitochondrial turbulence is a significant factor to induce apoptosis of cardiomyocytes, which is greatly involved in MH. A decrease in cardiomyocyte apoptosis contributes to the alleviation of MH.^[^ ^34^, ^35^, ^36^ ^]^ It has been reported that mitochondrial disorder‐dependent cardiomyocyte apoptosis exerts an in‐depth impact on MH genesis and progression.^[^ ^34^ ^]^ Also, other researchers have found that mitochondrial oxidative stress facilitates MH, subsequently fibrosis and remodeling.^[^ ^50^ ^]^ There are developed MH prevention and treatment strategies based on maintenance of mitochondrial homeostasis, including antioxidant MitoQ_10_^[^ ^51^ ^]^ and superoxide dismutase mimic EUK‐1340 for mitochondrial ROS elimination,^[^ ^52^ ^]^ Diazoxide for mitochondrial ATP‐sensitive potassium channel activation.^[^ ^53^ ^]^ As a cytoprotective factor, MANF has been found to have a potential anti‐hypertrophic effect in ischemic heart injury,^[^ ^24^ ^]^ and MANF silencing can worsen MH.^[^ ^54^ ^]^ Also, MANF acts as a regulatory factor for cardiac myocyte growth.^[^ ^55^ ^]^ The mechanism on how MANF is involved in MH is intricate. In this research, we explore the role of MANF in MH progress from the perspective of mitochondrial homeostasis and energy metabolism. Besides MANF, there are some other genes with significant changes after MH occurs, like TLR4. TLR4 up‐regulation has the potential to enhance TLR4/NF‐κB signaling and further worsen cardiac hypertrophy and fibrosis.^[^ ^56^ ^]^ Our previous report has verified an inhibitory role of MANF in TLR4/NF‐κB activation via MANF‐S100A8 interaction,^[^ ^18^ ^]^ indicating another possible channel for cardiomyocyte MANF to protect against MH through TLR4 suppression. Using KEGG Enrichment Analysis, dozens of DEGs are found to be closely associated with the PI3K/Akt signaling pathway, which is in line with previous reports that hypertrophic cardiac tissues often have an elevated PI3K/Akt activity.^[^ ^57^, ^58^ ^]^ It is possible that the increased myocardial MANF partly contributes to the intensive PI3K/Akt activity in MH, because MANF enables to activation PI3K/Akt signaling.^[^ ^57^ ^]^ Also, MANF‐mediated AKT phosphorylation results in nuclear factor erythroid 2‐related factor (Nrf2) nuclear translocation for anti‐oxidation and anti‐apoptosis,^[^ ^57^ ^]^ and this MANF‐Akt‐Nrf2 pathway may participate in cardiomyocyte MANF's resistance effects of mitochondrial oxidative stress and cardiac apoptosis in the MH pathological process. Also, in this study, we found cardiomyocyte PINK1/Parkin mitophagy induced by Ang II was partly inhibited after MANF vacancy. In consideration of the verified regulatory role of MANF in PINK1/Parkin mitophagy,^[^ ^41^ ^]^ it is possible that the promoting effect of MANF on mitophagy is another effective pathway involved in MANF‐mediated MH resistance, due to PINK1/Parkin mitophagy's protective function against Ang II‐induced cardiac cytotoxicity.^[^ ^40^ ^]^ These potential mechanisms of MANF's cardiac protection effects need to be further explored in the future.

Generally, glycolysis‐mediated energy supply is gradually enhanced along with the ever‐worsening cardiac injury and maladjustment.^[^ ^32^, ^59^ ^]^ In this study, we found that myocardial cell‐derived MANF knockout could further weaken mitochondrial oxidative phosphorylation, but enhance glycolysis and pyruvate/lactate production in pathological MH, accompanied by the increased oxidative phosphorylation‐associated enzymes like Cox family members. According to these findings, we speculate: under TAC or Ang II stimulation, MANF deficiency in myocardial cells exacerbates mitochondrial dysfunction to further impair oxidative phosphorylation in mitochondria and cause energy deficit of cardiomyocytes. Subsequently, anaerobic glycolysis and lactate biosynthesis in cardiomyocytes are greatly strengthened to respond to the insufficient ATP supply. Besides, the transcriptional expression of oxidative phosphorylation‐related factors is also enhanced to possibly act as a compensatory feedback for the disorder of oxidative phosphorylation and ATP generation. It has been reported that the up‐regulated Cox subunit antagonizes ATP deficiency in cardiomyocytes,^[^ ^60^ ^]^ and the long‐term chronic hypoxia causes an increase of Cox subunit mRNAs but an inhibition of Cox enzyme activity.^[^ ^61^ ^]^ For TAC‐triggered cardiomyocyte hypertrophy, the direct evidences from scRNA‐seq, WB, and qRT‐PCR prove that myocardial cell‐derived MANF deficiency leads to higher mRNA and protein levels of mitochondrial complex IV subunits; besides, the enzyme activity of complex I, II/III, and IV shows partial attenuation after MANF knockout. In view of the declining amount of acetyl‐CoA and the increased metabolites of glucose‐pyruvate‐lactate pathway, it is clear that myocardial cell‐specific MANF deficiency promotes ATP production‐oriented energy metabolism to be more partial to glucose‐lactate conversion, rather than mitochondrial oxidative phosphorylation. In terms of mechanism, the further imbalance of glycolysis and oxidative phosphorylation is largely due to the worse mitochondrial function and stability caused by MANF absence. We found that a lack of MANF could markedly reduce mitochondrial membrane potential (MMP) in both untreated and Ang II‐stimulated primary cardiocytes, and almost double Ang II‐induced mitochondrial ROS accumulation, indicating the intracellular MANF's substantial contribution to mitochondrial stability and energy metabolism.

A previous study has demonstrated C‐terminal domain (residues 96–158) of MANF is highly homologous to C‐terminal SAP domain (residues 563–608) of Ku70, including structural similarity, conserved lysines, and hydrophobic amino acids.^[^ ^44^ ^]^ Besides, Ku70 C‐terminal sequence and structure (residues 578–608) have been verified to be responsible for BAX binding and cytoplasmic retention.^[^ ^62^, ^63^ ^]^ Our SPR experimental results demonstrate that MANF's C‐terminal VKELKK motif is vital for BAX binding. BAX is an important regulatory protein for mitochondrial stability, and it translocates from cytoplasm to mitochondrial membrane, then promoting a release of cytochrome c and cardiomyocyte apoptosis.^[^ ^42^, ^45^ ^]^ As a consequence, BAX‐mediated mitochondrial damage and myocardial apoptosis further cause MH and other heart diseases.^[^ ^49^, ^64^, ^65^ ^]^ Zeng et al. find that Bisacurone inhibits BAX/Caspase‐3‐mediated apoptosis for alleviation of pressure overload‐induced cardiac hypertrophy.^[^ ^64^ ^]^ Also, through high‐throughput microscopy imaging and mRNA abundance analysis in cardiac myocytes stimulated by 15 hypertrophic agonists, BAX is found to have a high correlation with Ang II‐induced MH, and Ang II‐dependent BAX increase enables an acceleration of pathological cardiac hypertrophy to result in cardiac fibrosis and dysfunction.^[^ ^66^ ^]^ We proved the predicted protein interaction between MANF and BAX in this study, which was analogous to Ku70‐BAX binding. MANF could be regarded as a substitute BAX binding protein to impede BAX mitochondrial translocation and cytochrome c release under Ku70 acetylation‐triggered Ku70‐BAX dissociation,^[^ ^63^ ^]^ thereby playing a protective role against caspase‐dependent apoptosis. Maarit Hellman et al. have clearly confirmed that both the full‐length protein and C‐terminal peptide of MANF have a similar effect as Ku70 on neural protection.^[^ ^44^ ^]^ Our study here reveals that MANF is also able to protect cardiomyocytes against hypertrophy and death via BAX binding and mitochondria‐dependent apoptosis inhibition, and rhMANF administration partly mitigates more deteriorated MH in MKO mice. Differently, Mut C‐MANF loses its anti‐apoptosis capability in TAC or Ang II‐induced MH due to its incompetence for BAX binding. These findings indicate that rhMANF protein has the potential to be used as an effective therapy in MH medical intervention, especially for MH patients with low myocardial MANF expression.

In summary, our results in this study demonstrate a positive effect of MANF on cardiomyocyte protection and MH suppression, also reveal an underlying mechanism of how the intracellular MANF is involved in mitochondrial regulation and apoptosis resistance. Under the pathological state of cardiac hypertrophy, the binding of MANF to BAX can decrease BAX mitochondrial membrane translocation and cytochrome c release to cytoplasm, eventually relieving caspase‐dependent cardiomyocyte apoptosis. These findings not only give a potential clinical therapy or drug for MH treatment, but also further complete MANF's cell function regulatory network, especially cell death‐associated pathways. However, in this study, whether MANF's C‐terminal domain is responsible for BAX binding needs to be further verified in the future; also, the exogenous rhMANF exerts a protective effect against MH via entering cardiomyocytes, and we need more evidence to demonstrate the detailed channel for rhMANF entry intracellularly.

## 4 Experimental Section

### Patients

Human left ventricle tissues were collected from 10 MH patients and 10 healthy donors, respectively, at the First Affiliated Hospital of Anhui Medical University (Hefei, China), which were divided into the healthy control (CON) group (*n* = 10) and myocardial hypertrophy (MH) group (*n* = 10). Human serum samples were collected from 80 MH patients and 30 healthy volunteers respectively for CON group (*n* = 30) and the MH group (*n* = 80). Diabetes mellitus, infective endocarditis, active rheumatism, pulmonary disease, hyperthyroidism, autoimmune diseases, and others were not found in all patients and donors. MH‐related diagnostic data and other demographic information of MH Patients and CON Healthy groups were compared in Tables S1 and S2 (Supporting Information), respectively. All the experiments related to human tissues were performed following the Helsinki Declaration. The research protocol was approved by the Human Ethics Committee of the First Affiliated Hospital of Anhui Medical University (Approval number: PJ2023‐02‐17). Written informed consent was obtained from all involved patients and donors.

### Mice

MANF conditional knockout mice were *Manf* ^flox/flox^ C57BL/6 mice bearing loxP sites flanking exons 3 of *manf* gene, which were provided by Professor Jia Luo from the University of Kentucky as a gift. Myocardial cell‐specific MANF knockout (MKO) mice were generated by using MANF conditional knockout mice and Myh6‐cre mice for mating. The littermate *Manf* ^flox/flox^ C57BL/6 mice were involved as wild‐type (WT) mice. All animal experiments were performed according to protocols approved by the Animal Ethics Committee of Anhui Medical University (Approval number: LLSC20200022). All animal procedures conform to the NIH Guide for the Care and Use of Laboratory Animals. Please refer to more experimental details in the Supporting Information.

### TAC or Ang II‐Induced Myocardial Hypertrophy Mice Model

The MH mice model was established by TAC or Ang II treatment of MKO and WT male mice at 9 weeks old. For the TAC‐induced MH mice model, mice were used for cardiac surgery to expose aortic arch, followed by aortic arch ligation with moderate tightness (Aortic dimension: ≈0.4 mm). For Ang II‐induced MH mice model, mice were used for skin incision to subcutaneously implant micro Ang II pump (Ang II dosage: 1000 ng kg min^−1^). The Sham group was produced by the same cardiac surgery without aortic arch ligation for TAC control or subcutaneous implantation of a micro normal saline pump for Ang II control. Please refer to more experimental details in the Supporting Information.

### Primary Cardiocytes Isolation and Culture

Primary cardiocytes were isolated and cultured according to the previous report.^[^ ^67^ ^]^ Briefly, inhalational anesthesia was conducted by isoflurane evaporator of an anesthesia machine (Isoflurane gas dose: 4% for anesthesia induction, 1.5% for anesthesia maintenance; flow rate: 1 L min^−1^, details in the following part of anesthesia and sacrifice). Mice were sacrificed by heart perfusion using Langendorff perfusion system. Hearts were removed from mice. Calcium‐free perfusate was first used for 5 min, then Krebs‐Henseleit Bicarbonate (KHB) Buffer with collagenase II (1 mg mL^−1^) and hyaluronidase was involved in heart perfusion for 15 min. Primary cardiocytes were collected in DMEM/F12 medium with Glutathione (10 mmol L^−1^), Sodium bicarbonate (26.2 mmol L^−1^), 20% Foetal Bovine Serum (FBS), Penicillin (100 U mL^−1^) and Streptomycin (100 µg mL^−1^), and cultured on cell‐culture dishes or glass coverslips coated with Laminin (20 µg mL^−1^). Primary cardiocytes from WT and MKO mice were treated by Ang II (0.2 µm) for 48 h in vitro.

### Sirius Red and Masson Staining

Paraffin sections of cardiac tissues from humans and mice were used for Sirius red and Masson staining according to the previous report.^[^ ^18^ ^]^ Briefly, deparaffinization was performed, followed by rehydration in 100%, 90%, 80% and 70% ethanol. For Sirius red staining, Sirius red staining kit (Solarbio, Beijing, China, G1472) was used to stain for 1 h, then rinsed for 30 min. For Masson staining, Masson staining kit (Solarbio, G1346) was used. Please refer to more experimental details in the Supporting Information. Images were obtained by Olympus Microscope BX53/IX71.

### Immunohistochemistry (IHC)

Immunohistochemistry was performed on cardiac tissues from humans and mice according to the previous report.^[^ ^18^ ^]^ After 10% formaldehyde fixation and paraffin embedding, paraffin sections were used for deparaffinization in dimethylbenzene, followed by rehydration in 100%, 90%, 80% and 70% ethanol. For hematoxylin‐eosin (HE) staining, hematoxylin staining was first performed for 6 min, followed by rinsing for 15 min. Then, eosin staining was performed for 3 s, followed by rinsing for 5 min. Paraffin sections were placed in dimethylbenzene for 40 min. For immunohistochemical staining, paraffin sections were stained by MANF antibody for 2 h at 37 °C, followed by 3, 3′‐diaminobenzidinetetrahydrochloride (DAB) staining and hematoxylin counterstaining. Images were obtained and analyzed by Olympus Microscope BX53 and cellSens Standard software.

### Western Blot (WB)

Protein samples in cell lysates were involved in the reduced sodium dodecyl sulfate polyacrylamide gel electrophoresis (SDS‐PAGE) for protein separation. 10 µg total protein for each sample was used. Then, proteins were transferred to a PVDF membrane. 5% BSA was used for blocking, followed by primary antibody incubation. After secondary antibody incubation, the results were visualized by Chemiscope 6000 Pro Touch imaging system. Please refer to the primary antibodies’ details in the Supporting Information.

### Quantitative Real Time Polymerase Chain Reaction (qRT‐PCR)

Trizol reagent was used for total RNA extraction, then PrimeScript RT reagent kit (TaKaRa Bio, Dalian, China, RR047A) was involved in reverse transcription according to the manufacturer's instructions. Please refer to the primers’ details in the Supporting Information.

### Enzyme‐Linked Immunosorbent Assay (ELISA)

Serum samples from humans and mice were used for ELISA of MANF level according to the manufacturer's instruction. The utilized ELISA kit was Mouse MANF ELISA Kit (Cloud‐Clone Corp, Wuhan, China, SEC300Mu).

### Immunofluorescent Staining

Immunofluorescent staining was performed by mice's cardiac tissues and primary cardiocytes cultured in vitro according to the previous report.^[^ ^18^ ^]^ For paraffin sections of cardiac tissues, deparaffinization was performed, followed by rehydration in 100%, 90%, 80% and 70% ethanol. For primary cardiocytes, cell culture slides were prepared first. Paraffin sections and cell culture slides were incubated with 5% BSA, then antibodies for 2 h at 37 °C. DAPI was used to stain nuclei for 10 min. Images were obtained and analyzed by Olympus Microscope BX53 and cellSens Standard software.

### Terminal Deoxynucleotidyl Transferase dUTP Nick end Labeling (TUNEL) Assay

Mice's cardiac tissues were used for TUNEL assay by In Situ Cell Death Detection Kit (Roche, Basel, Switzerland, 11684795910) according to the manufacturer's instruction. The images were collected by Olympus Microscope BX53/IX71.

### High‐Throughput Proteomic Screening

WT mice in Sham and TAC treatment groups were used for high‐throughput proteomic screening, which were divided into Sham group (eight mice, *n* = 8) and TAC group (eight mice, *n* = 8), respectively. Please refer to more experimental details in the Supporting Information.

### Single‐Cell RNA Sequencing (scRNA‐seq)

WT and MKO mice with TAC treatment were used for scRNA‐seq. Cardiac tissues from six WT (*n* = 6) and MKO (*n* = 6) mice were collected to mix every three tissue samples into one for a total of two WT and two MKO tissue mixtures, respectively, which were further used for single‐cell isolation. Please refer to more experimental details in the Supporting Information.

### Metabolomics Analysis

WT and MKO mice with TAC treatment were used for metabolomics analysis. Six WT and MKO mice were divided into the WT group (*n* = 6) and the MKO group (*n* = 6), respectively. Please refer to more experimental details in the Supporting Information.

### Oxygen Consumption Rate and Extracellular Acidification Rate Assay

WT and MKO primary cardiocytes with Ang II (0.2 µm) and rhMANF (20 µg) treatment in vitro, as indicated, were involved in Oxygen Consumption Rate (OCR) and Extracellular Acidification Rate (ECAR) assays performed by Agilent Seahorse XF Cell Mito Stress Test Kit (Agilent, CA, USA, 103015–100) and XF Glycolysis Stress Test Kit (Agilent, 103020–100) according to the manufacturer's instruction. Briefly, primary cardiocytes (2 × 10^4^ cells) were cultured in 96‐well Agilent Seahorse XF Cell Culture Microplate. After incubation overnight, the culture medium was replaced, followed by incubation in CO_2_‐free and 37 °C environment for 1 h. Then, OCR and ECAR were measured according to the procedure.

### Transmission Electron Microscopy (TEM) Scanning

WT and MKO primary cardiocytes with or without Ang II (0.2 µm) treatment for 48 h in vitro were used for TEM scanning. For sample preparation, the cultured cardiocytes were treated with TEM fixing agent (Servicebio, G1102) for 4 h, followed by centrifugation, 1% agarose treatment, and phosphate buffer (0.1 m, pH 7.4) washing. Then, 1% osmic acid was involved in fixation for 2 h, followed by phosphate buffer washing and dehydration in 50%, 70%, 80%, 90%, 95%, 100% ethanol and 100% acetone. After permeation, embedding, and ultrathin section (60–80 nm), dual dyeing of 2% uranyl acetate solution and lead citrate was performed. TEM images were obtained by HITACHI HT7700 transmission electron microscopy.

### Co‐immunoprecipitation

Primary cardiocytes and AC16 cells were used to produce cell lysates by IP lysis buffer (50 mm HEPES, pH 7.4; 150 mm NaCl; 0.1% Triton X‐100; 2 mM EGTA; protease inhibitors). Co‐immunoprecipitation (Co‐IP) was performed according to the previous report.^[^ ^18^ ^]^ First, protein A/G plus‐agarose precleaning was performed, followed by incubation with Anti‐MANF (Abcam, ab67271) or Anti‐BAX (Abcam, ab182733) overnight at 4 °C. Then, protein A/G plus‐agarose was added, and Co‐IP washing buffer (20 mm Tris‐HCl, pH 7.6; 100 mm NaCl; 1 mm EDTA) was used for washing. The results were visualized by SDS‐PAGE and immunoblotting.

### Flow Cytometry

WT and MKO primary cardiocytes with Ang II (0.2 µm) and rhMANF (20 µg) treatment in vitro, as indicated, were used for flow cytometry (FCM). First, cell suspensions were produced, followed by blocking in 1% rat serum. Staining was performed by incubation with Annexin V‐FITC, TMRM‐PE, MitoTracker‐FITC or MitoSOX‐PE, respectively. For Annexin V‐FITC/PI apoptosis, the PI staining solution was used for PI staining. After washing, cell suspensions were detected and analyzed by BD FACS Verse.

### Cell Fractionation

Mitochondrial and cytoplasmic proteins were extracted by cell fractionation according to the previous report.^[^ ^68^ ^]^ WT and MKO primary cardiocytes with or without Ang II (0.2 µm) treatment for 48 h in vitro were washed with Ca^2+^ and Mg^2+^‐free PBS buffer, followed by incubation in pre‐cooling hypotonic solution (25 mm HEPES, pH 7.5; 5 mm MgCl_2_; 1 mm EGTA; 1 mm EDTA; 1 mm sodium vanadate; 20 nm microcystin; protease inhibitors). Cell lysates were produced by homogenization, followed by 800 × g centrifugation for 15 min to obtain supernatant. After that, the collected supernatant was centrifuged at 4500 × g for 15 min to obtain the pellet for mitochondrial proteins and the supernatant for cytoplasmic proteins.

### Surface Plasmon Resonance

A Biacore assay was performed for SPR detections by Biacore X100 biomolecular interaction analysis system (Biacore, GE Healthcare). BAX as the ligand protein, was diluted by sodium acetate (10 mm) to 50 µg mL^−1^, then coupled on CM5 Sensor Chips (Cytiva, BR‐1005‐30). The flow rate was set as 10 µL min^−1^. MANF and Mut C‐MANF were involved in protein interaction analysis respectively. 1 × PBS‐P + solution (Cytiva, 28‐9950‐84, 5% DMSO, pH 7.4) was used as running buffer with the flow rate of 30 µL min^−1^ and a duration of 80 s, followed by regenerating the chip via 10 mM glycine hydrochloride solution (pH 2.0).

### 13C Metabolic Flux Analysis

^13^C glucose tracing in vitro was performed according to the previous report.^[^ ^69^ ^]^ Briefly, glucose‐free DMEM medium was used to produce ^13^C‐labelled medium containing 50% U‐^13^C‐Glucose (Targetmol Chemicals, MA, USA, T19261) and 50% unlabelled ^12^C‐Glucose. WT and MKO primary cardiocytes (5 × 10^6^ cells) were collected for culturing in ^13^C‐labelled medium, along with Ang II (0.2 µm) treatment for 48 h. Then, primary cardiocytes were collected for LC‐MS analysis by Thermo Q Exactive PLUS hybrid quadrupole‐orbitrap mass spectrometer coupled with hydrophilic interaction chromatography.

### Mitochondrial Complex Activity Assay

WT and MKO primary cardiocytes in TAC‐induced MH mice were used for mitochondria isolation, followed by mitochondrial complex activity examination according to the manufacturer's instructions. Complex I Enzyme Activity Assay Kit (Abcam, ab109721), MitoTox Complex II+III OXPHOS Activity Assay Kit (Abcam, ab109905), and Complex IV Rodent Enzyme Activity Microplate Assay Kit (Abcam, ab109911) were used for activity detections of Complex I, Complex II/III, and Complex IV, respectively. 100 µg total protein was involved in immunocapture of Complex I, II/III, and IV respectively, followed with Complex I activity evaluation via the rate of oxidation from NADH to NAD^+^, Complex II/III activity evaluation via the conversion of oxidized Cyt‐c into reduced form, Complex IV activity evaluation via the oxidation of reduced Cyt‐c and absorbance detection at 550 nm.

### Statistical Analysis

The integral optical density (IOD), Sirius red^+^ and collagen^+^ areas were calculated by Image J. Data were expressed as mean ± SD. GraphPad Prism software (v. 9.0a; GraphPad Software, La Jolla, CA, USA) was involved in data analysis. Two‐tailed Student's *t*‐test was used to calculate statistical significance between two groups. Two‐way ANOVA with Tukey's *post hoc* test was used to calculate statistical significance among multiple groups (≥3 groups). For a significant difference, *p* < 0.05 indicates a significant difference for one asterisk (*). *p* < 0.01 for two asterisks (**), *p* < 0.001 for three asterisks (***). Data are representative of three independent experiments.

## Conflict of Interest

The authors declare that they have no competing interests.

## Author Contributions

D.W., X.R.Z., and B.L.W. contributed equally to this work. C.H., C.H.W., and D.W. contributed to conceptualization, formal analysis, and project administration, acquired fund, and wrote the original draft. C.H., D.W., and X.R.Z. supervised the project, contributed to methodology, and data curation. B.L.W., and H.P.L. performed data curation, methodology, acquired resources, wrote, reviewed & edited the final manuscript. X.R.Z., B.L.W., H.P.L., D.S.X., Y.Y., J.L.Z., W.B.W., R.Z., X.Y.W., Y.F.C., and S.Y.C. contributed to data curation and methodology.

## Supporting information



Supporting Information

## Data Availability

All the datasets analyzed in this study are available from the following publicly available repositories: scRNA‐seq and RNA‐seq datasets deposited in NCBI Sequence Read Archive (SRA) with accession numbers of SRR29025683, SRR29025682, SRR29025681, SRR29025680, and SRR32205132; proteomic dataset deposited in iProX with ProteomeXchange ID of PXD052275; metabolomics dataset deposited in Genome Sequence Archive (GSA) with OMIX ID of OMIX006466 and OMIX008636.
