# Supplementary material for: Mesencephalic Astrocyte‐Derived Neurotrophic Factor Binds BAX to Preserve Mitochondrial Homeostasis and Energy Metabolism for Relieving Myocardial Hypertrophy
Source: Adv Sci (Weinh). 2025 Jul 30;12(40):e02835. doi: 10.1002/advs.202502835 (PMC12561191; doi:10.1002/advs.202502835)
Supplement: Supplementary file 1 — Supporting Information [file ADVS-12-e02835-s001.docx]

**Supporting Information**

Mesencephalic Astrocyte-derived Neurotrophic Factor Binds BAX to Preserve Mitochondrial Homeostasis and Energy Metabolism for Relieving Myocardial Hypertrophy

Dong Wang, Xinru Zhang, Baolong Wang, Haipeng Li, Dongshuo Xu, Yun Yang, Jialu Zhang, Wenbing Wang, Ren Zhang, Xinyu Wang, Yunfeng Cai, Shiyu Cao, Chao Hou,* and Changhui Wang*

Anhui Medical University, Hefei 230032, Anhui, China; The First Affiliated Hospital of Anhui Medical University, Hefei 230022, Anhui, China

E-mail: [wangchanghui@ahmu.edu.cn](mailto:wangchanghui@ahmu.edu.cn); [houchao@ahmu.edu.cn](mailto:houchao@ahmu.edu.cn); [wangdong@ahmu.edu.cn](mailto:wangdong@ahmu.edu.cn)

**Table of content**

Table S1……………………………………………………………………………….3

Table S2……………………………………………………………………………….4

Figure S1………………………………………………………………………………5

Figure S2………………………………………………………………………………7

Figure S3………………………………………………………………………………9

Figure S4……………………………………………………………………………..11

Figure S5……………………………………………………………………………..13

Figure S6……………………………………………………………………………..15

Figure S7……………………………………………………………………………..17

Figure S8……………………………………………………………………………..19

Figure S9……………………………………………………………………………..21

Figure S10……………………………………………………………………………23

Figure S11……………………………………………………………………………25

Experimental Details…………………………………………………………………27

Table S1. Heart-related diagnostic information of MH Patients Group and CON Healthy Group

| Item | MH Patients (*n*=80) | CON Healthy (*n*=30) | *p* value |
| --- | --- | --- | --- |
| BNP [pg/ml, M (Q1, Q3)] | 65.74 (27.26, 279.13) | 20.90 (11.5, 39.96) | <0.001 |
| hs-Tn [ng/ml, M (Q1, Q3)] | 0.01 (0.001, 0.008) | 0.001 (0.001, 0.001) | <0.001 |
| LVEDD [cm, M (Q1, Q3)] | 5.02 (4.70, 5.35) | 4.65 (4.32, 4.76) | <0.001 |
| IVST [cm, M (Q1, Q3)] | 1.21 (1.15, 1.28) | 0.91 (0.86, 1.00) | <0.001 |
| LVPWD [cm, M (Q1, Q3)] | 1.02 (0.94, 1.07) | 0.88 (0.82, 0.94) | <0.001 |
| LVEF [%, M (Q1, Q3)] | 60.0 (54.5, 62.0) | 63.0 (60.0, 66.0) | <0.001 |
| R_V5_+S_V1_ [mv, M (Q1, Q3)] | 2.13 (1.55, 3.01) | 1.85 (1.37, 2.09) | 0.023 |
| QRS Wave Duration  [ms, M (Q1, Q3)] | 92.50 (84.0, 102.0) | 88.0 (84.0, 96.0) | 0.038 |

BNP: Brain Natriuretic Peptide; hs-Tn: High Sensitivity Troponin; LVEDD: Left Ventricular End-Diastolic Diameter; IVST: Interventricular Septal Thickness; LVPWD: Left Ventricular Posterior Wall Thickness; LVEF: Left Ventricular Ejection Fraction. Two-tailed Student’s t-test is used for *p* value calculation.

Table S2. Demographic and other clinical information of MH Patients Group and CON Healthy Group

| Item | MH Patients (*n*=80) | CON Healthy (*n*=30) | *p* value |
| --- | --- | --- | --- |
| Age [Years, x±s]  Gender [Male/Female, %]  BMI [kg/m^2^] | 62.5±15.8  54/26 (67.5%/32.5%)  26.2±3.3 | 53.6±10.3  21/9 (70%/30%)  25.5±3.2 | <0.01  n.s.  n.s. |
| Overweight [n, %] | 65 (81.25%) | 23 (76.67%) | n.s. |
| DLP [n, %] | 48 (60%) | 17 (56.67%) | n.s. |
| Diabetes [n, %] | 0 (0%) | 0 (0%) | NA |
| IE [n, %] | 0 (0%) | 0 (0%) | NA |
| Rheumatism [n, %] | 0 (0%) | 0 (0%) | NA |
| COPD [n, %] | 0 (0%) | 0 (0%) | NA |
| Hyperthyroidism [n, %] | 0 (0%) | 0 (0%) | NA |
| AD [n, %] | 0 (0%) | 0 (0%) | NA |

BMI: Body Mass Index; DLP: Dyslipidemia; IE: Infective Endocarditis; COPD: Chronic Obstructive Pulmonary Disease; AD: Autoimmune Diseases. Two-tailed Student’s t-test is used for *p* value calculation. NA, Not applicable; n.s., No significance.


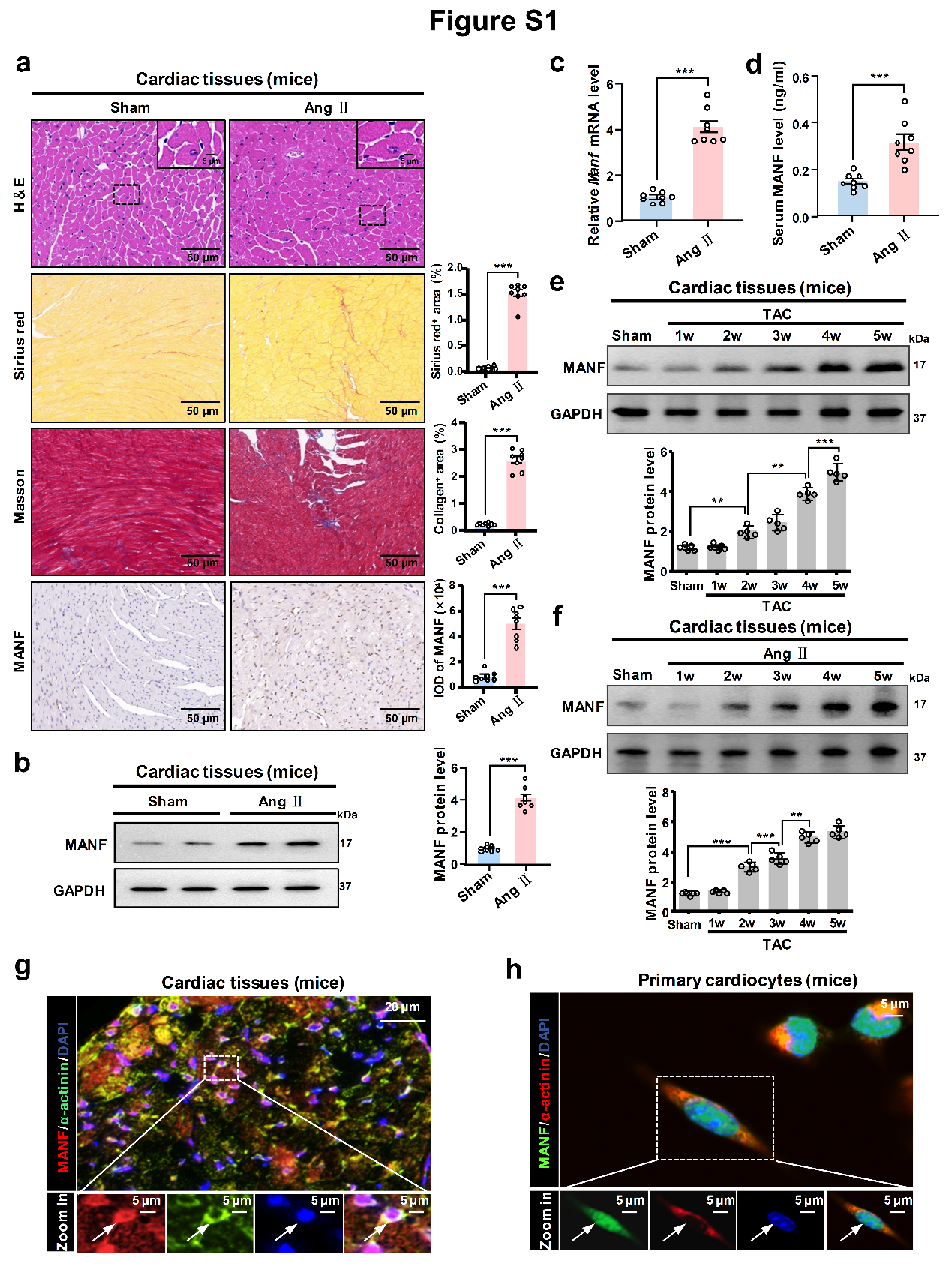


**Figure S1.** MANF expression was significantly enhanced in cardiac tissues and serum samples of Ang II-induced MH mice. At the third week after Ang II injection, cardiac tissues and serum samples were collected from 8 Sham mice (n=8) and 8 Ang II mice (n=8) respectively. a) HE, Sirius red, Masson staining and MANF immunohistochemistry were performed. Sirius red positive area, collagen positive area and integral optical density of MANF were calculated (n=8). Scale bar: 50 or 5 μm. b) WB and c) qRT-PCR (n=8) were performed to evaluate MANF protein and mRNA levels. d) ELISA (n=8) was performed to examine the serum MANF level. MANF expression in TAC e) and Ang II f)-induced MH cardiac tissues was monitored by WB at different time points of 1st, 2nd, 3rd, 4th, 5th week respectively. Immunofluorescent double labelled staining of MANF and α-actinin was performed in WT cardiac tissues g) and primary cardiocytes h) respectively. DAPI (Blue) was used for nuclei staining. Scale bar: 20 or 5 μm. Data are expressed as mean ± SD. ** p<0.01, *** p<0.001. Sham: sham operation; TAC: transverse aortic constriction; Ang II: angiotensin II. Data are representative of three independent experiments.


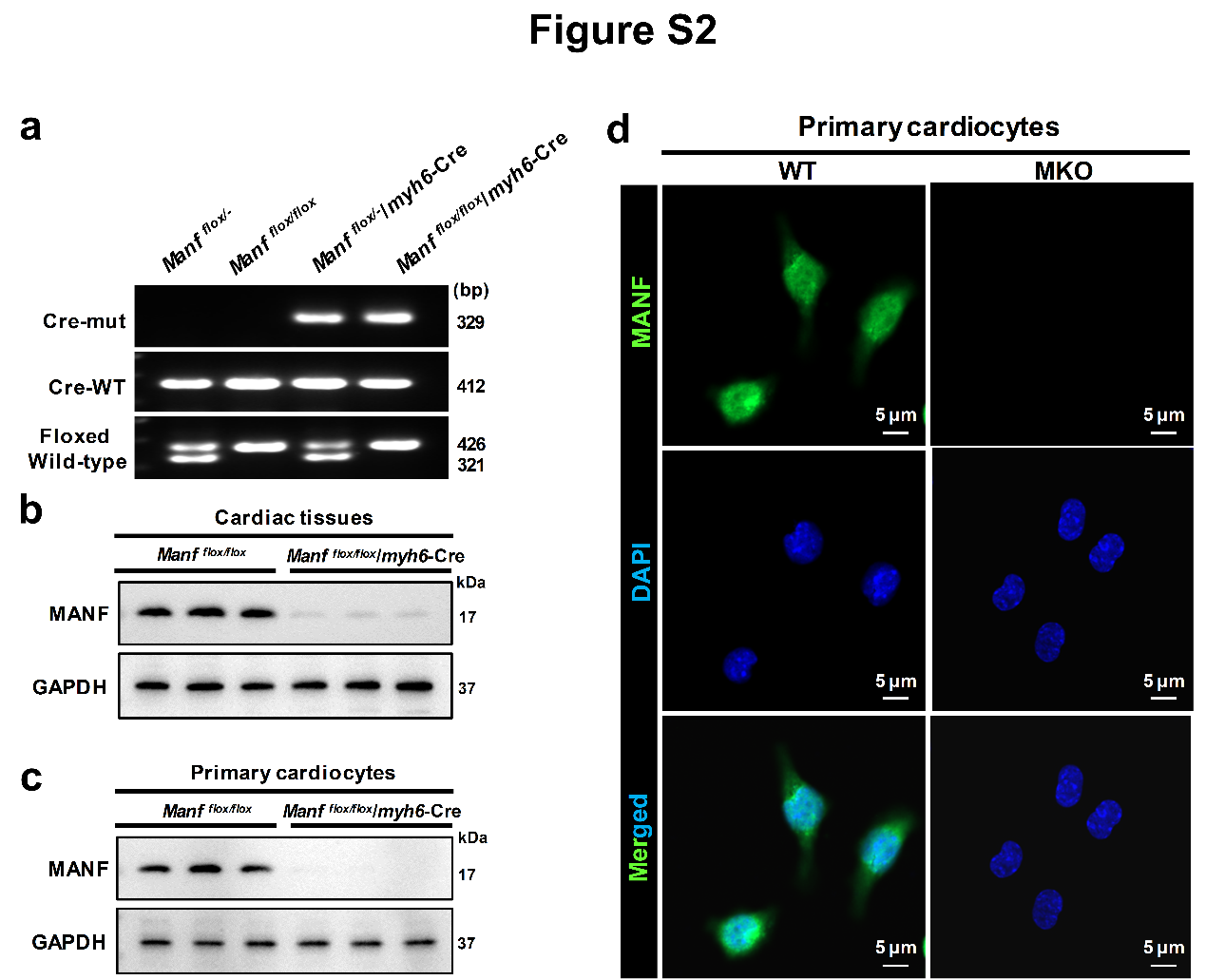


**Figure S2.** Identification of myocardial cell-specific MANF knockout mice. a) PCR analysis for myocardial cell-specific MANF knockout (n=3). WB analysis (n=3) for myocardial cell-specific MANF knockout by cardiac tissues b) and primary cardiocytes c). d) Immunofluorescent staining of MANF (Green) was performed in WT and MKO primary cardiocytes. DAPI (Blue) was used for nuclei staining. Scale bar: 5 μm. WT: wild type; MKO: myocardial cell-specific MANF knockout. Data are representative of three independent experiments.


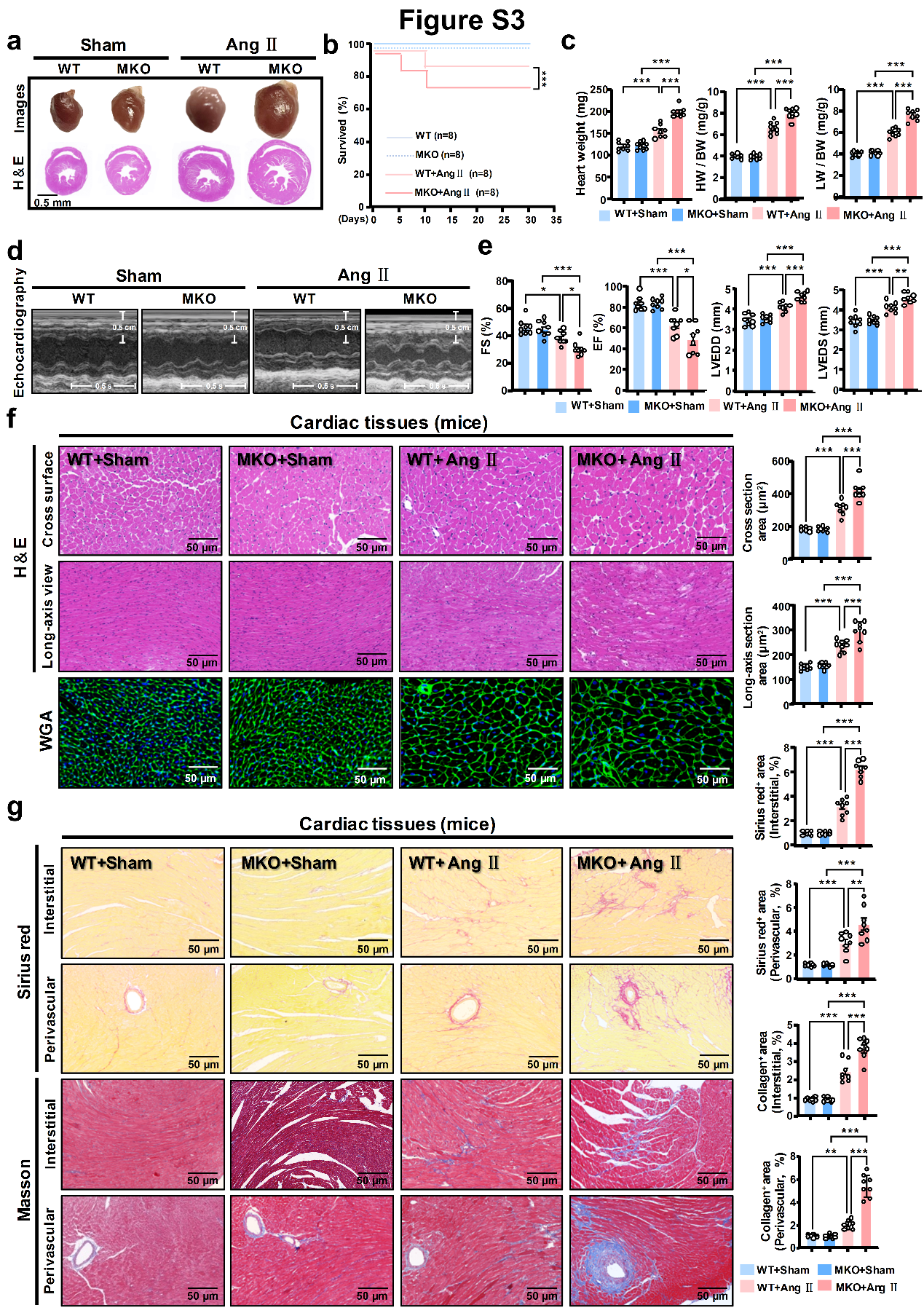


**Figure S3.** Myocardial cell-derived MANF alleviated Ang II-induced myocardial hypertrophy and cardiac dysfunction. WT and MKO mice were used to construct Ang II-induced MH model for Sham-WT (n=8), Sham-MKO (n=8), Ang II-WT (n=8) and Ang II-MKO (n=8) groups. At the third week after Ang II injection, cardiac tissues were collected. a) Heart images were collected and HE staining of cardiac tissues was performed. Scale bar: 0.5 mm. b) Mice’s survival rate was recorded once every five days. c) Heart weight (HW), lung weight (LW) and body weight (BW) were calculated respectively for ratios of HW/BW and LW/BW (n=8). d, e) Cardiac function was monitored by echocardiography (n=8) at the third week after Ang II injection. Cardiac tissues (n=8) were used for HE and WGA staining f), Sirius red and Masson staining g). HE staining included cross surface and long-axis view. Sirius red and Masson staining included interstitial and perivascular views. Cross section and long-axis section areas of HE staining, Sirius red positive area and collagen positive area were calculated. Scale bar: 50 μm. Data are expressed as mean ± SD. * p<0.05, ** p<0.01, *** p<0.001. Sham: sham operation; Ang II: angiotensin II; WT: wild type; MKO: myocardial cell-specific MANF knockout. Data are representative of three independent experiments.


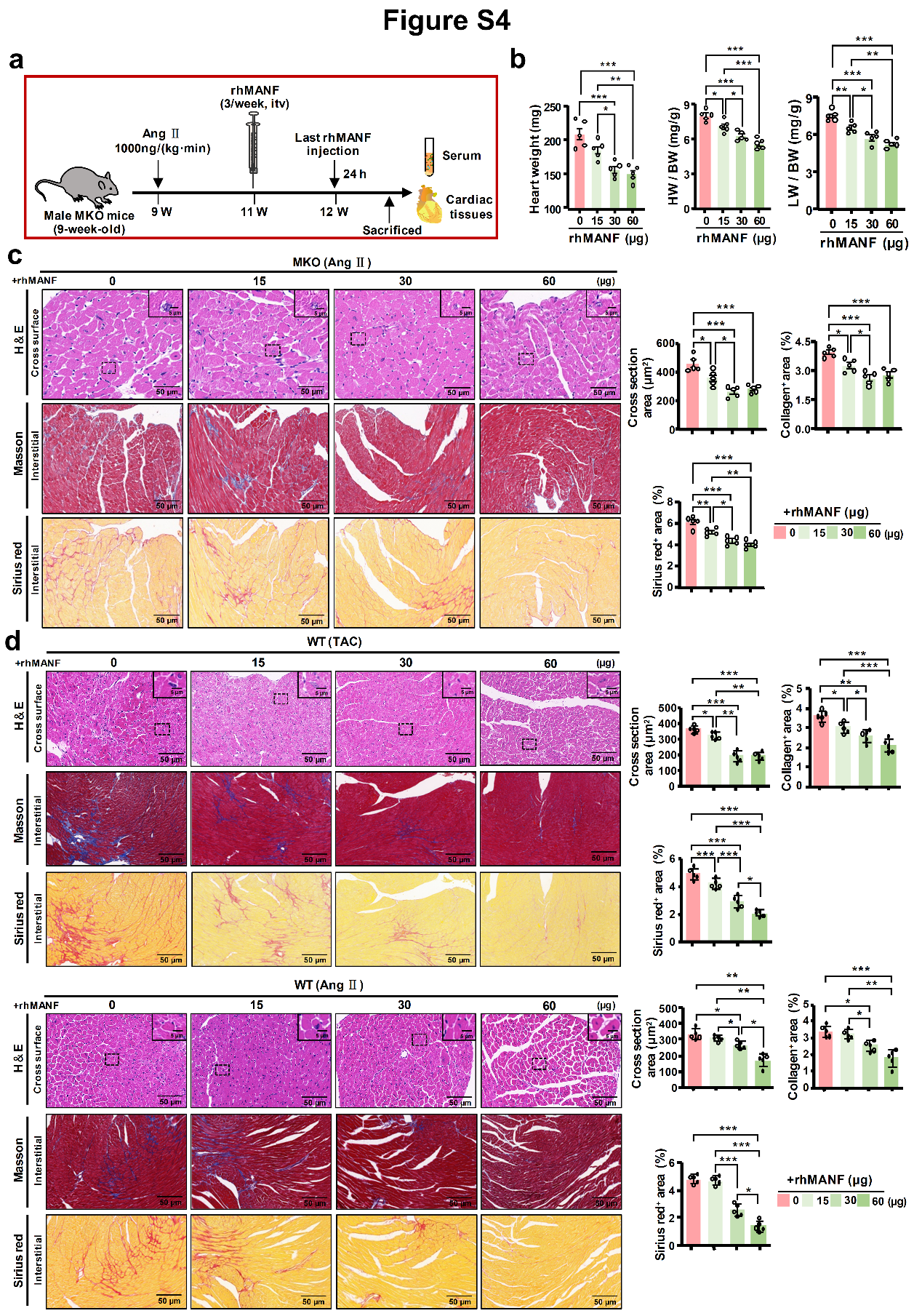


**Figure S4.** Recombinant human MANF protein relieved TAC or Ang II-induced myocardial hypertrophy in mice. MKO mice were used to construct Ang II-induced MH model, followed by rhMANF (0, 15, 30, 60 µg) treatment (n=5). a) Diagram of the procedure of Ang II-induced MH mice treated by rhMANF. At the second week after Ang II injection, rhMANF was intravenously injected three times per week. b) Heart weight (HW), lung weight (LW) and body weight (BW) were calculated respectively for ratios of HW/BW and LW/BW (n=5). c) Cardiac tissues (n=5) were used for HE (cross surface), Sirius red (interstitial view) and Masson (interstitial view) staining. d) WT mice were used to construct TAC or Ang II-induced MH model, followed by rhMANF (0, 15, 30, 60 µg) treatment (n=5). Cardiac tissues (n=5) were used for HE, Sirius red and Masson staining. Cross section area of HE staining, Sirius red positive area and collagen positive area were calculated. Scale bar: 50 or 5 μm. Data are expressed as mean ± SD. * p<0.05, ** p<0.01, *** p<0.001. TAC: transverse aortic constriction; Ang II: angiotensin II; WT: wild type; MKO: myocardial cell-specific MANF knockout; rhMANF: recombinant human MANF. Data are representative of three independent experiments.


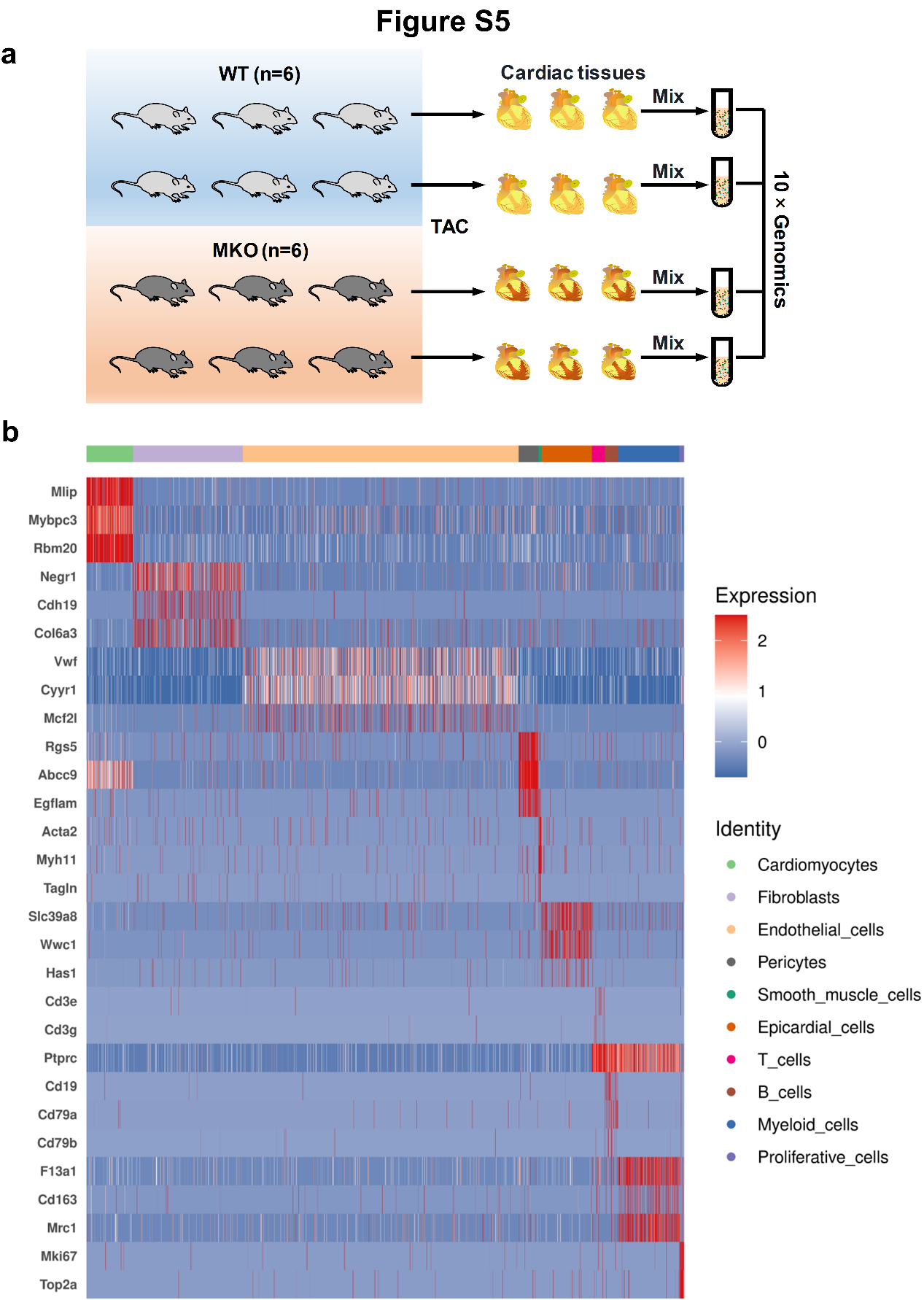


**Figure S5.** Single cell-RNA sequencing for TAC-induced MH model in WT and MKO mice. WT and MKO mice were used to construct TAC-induced MH model for TAC-WT (n=6) and TAC-MKO (n=6) groups, followed by scRNA-seq analysis. a) Diagram of WT and MKO cardiac tissue sample preparation for scRNA-seq (n=6). b) Heatmap associated with identification of different cell types. Cell type identification-related genes are listed longitudinally. Different colors horizontally indicate different cell types. TAC: transverse aortic constriction; WT: wild type; MKO: myocardial cell-specific MANF knockout.


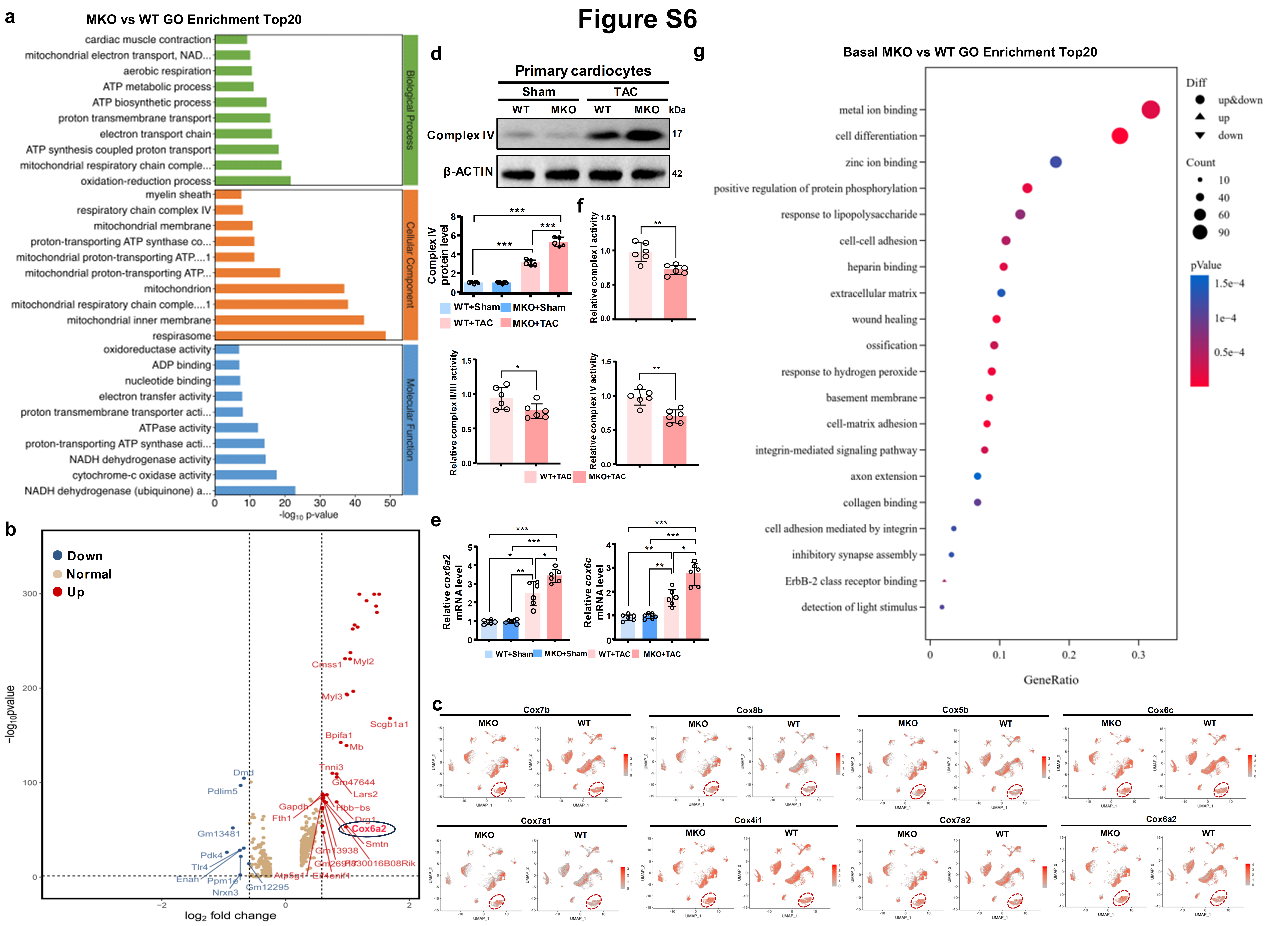


**Figure S6.** scRNA-seq analysis of DEGs and Cox gene family, mitochondrial complex expression and activity detections for TAC-induced myocardial hypertrophy in WT and MKO mice. WT and MKO cardiac tissue samples with TAC-induced myocardial hypertrophy (n=6) were prepared for scRNA-seq analysis. a) Analysis of DEGs-related top20 GO enrichment pathways. b) Volcano plot displaying DEGs between WT and MKO myocardial cells. Red, blue and brown dots represent genes with significant up-regulation, significant down-regulation and no significant difference respectively. c) Feature plots showing the representative Cox family genes in myocardial cells. WT and MKO primary cardiocytes from Sham and TAC-induced MH mice were used to examine complex IV protein level by WB d), mRNA levels of COX6A2 and COX6C by qRT-PCR e), relative activity of mitochondrial complex I, II/III and IV f). g) The normal WT and MKO primary cardiocytes (n=3) were prepared for RNA-seq analysis. DEGs-related top20 GO enrichment pathways were analyzed. The size and color of circles represents gene enrichment count and significance respectively. * p<0.05, ** p<0.01, *** p<0.001. Sham: sham operation; TAC: transverse aortic constriction; WT: wild type; MKO: myocardial cell-specific MANF knockout. Data are representative of three independent experiments.


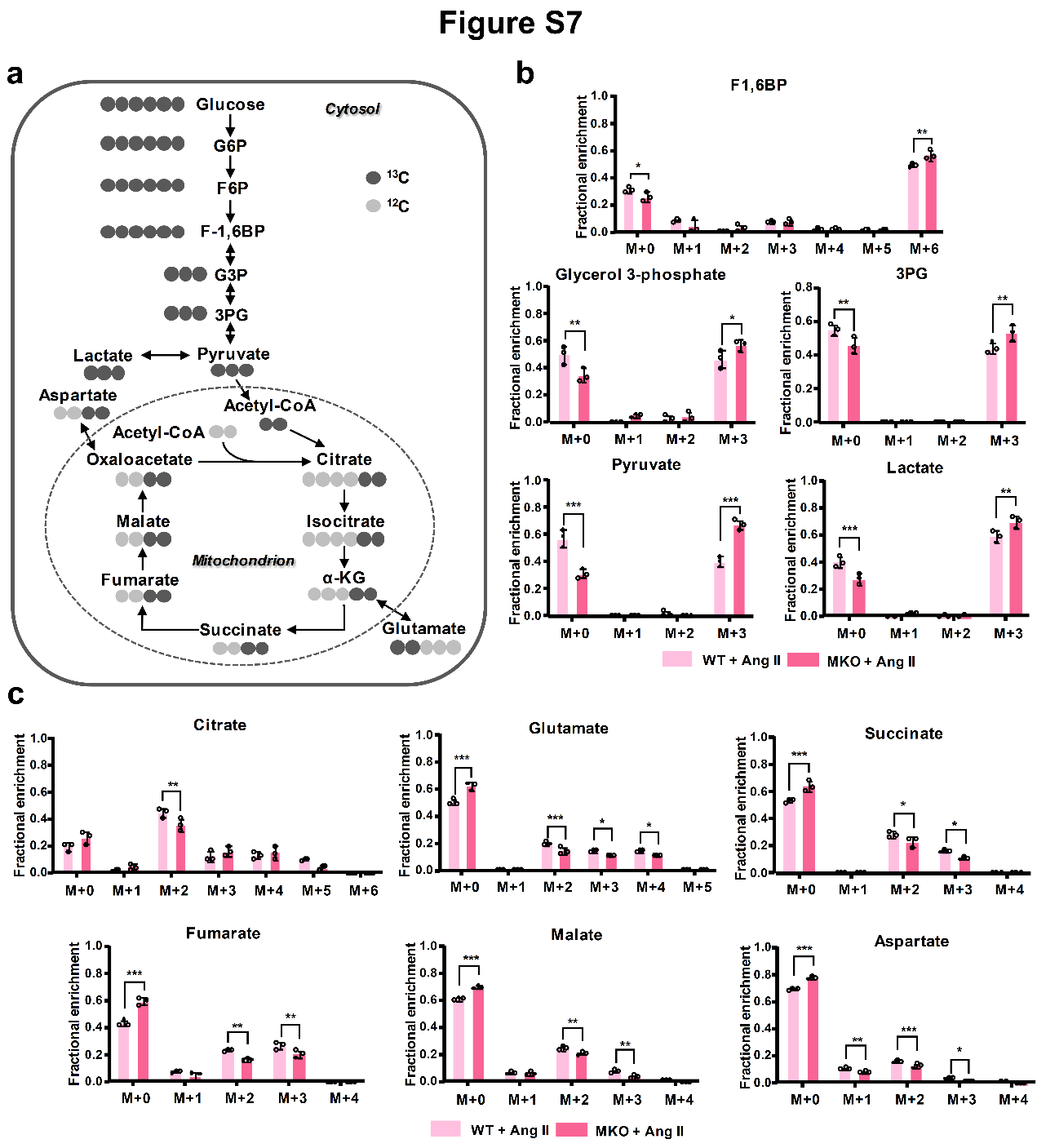


**Figure S7.** ^13^C Metabolic Flux Analysis of WT and MKO primary cardiocytes under Ang II-induced myocardial hypertrophy. WT and MKO primary cardiocytes (5×10^6^ cells) with Ang II (0.2 μM) treatment (n=3) were cultured in ^13^C-labelled medium containing 50% U-^13^C-Glucose and 50% unlabelled ^12^C-Glucose, followed by LC-MS analysis. a) Schematic of carbon involved into glycolysis and TCA cycle intermediates from U-^13^C-Glucose. Dark grey: ^13^C; Light grey: ^12^C. Fractional enrichment and labeling patterns of glycolytic b) and TCA cycle c) intermediates from U-^13^C-Glucose-labeled medium (n=3). Natural abundance correction was performed. Data are expressed as mean ± SD. * p<0.05, ** p<0.01, *** p<0.001. Ang II: angiotensin II; WT: wild type; MKO: myocardial cell-specific MANF knockout. Data are representative of three independent experiments.


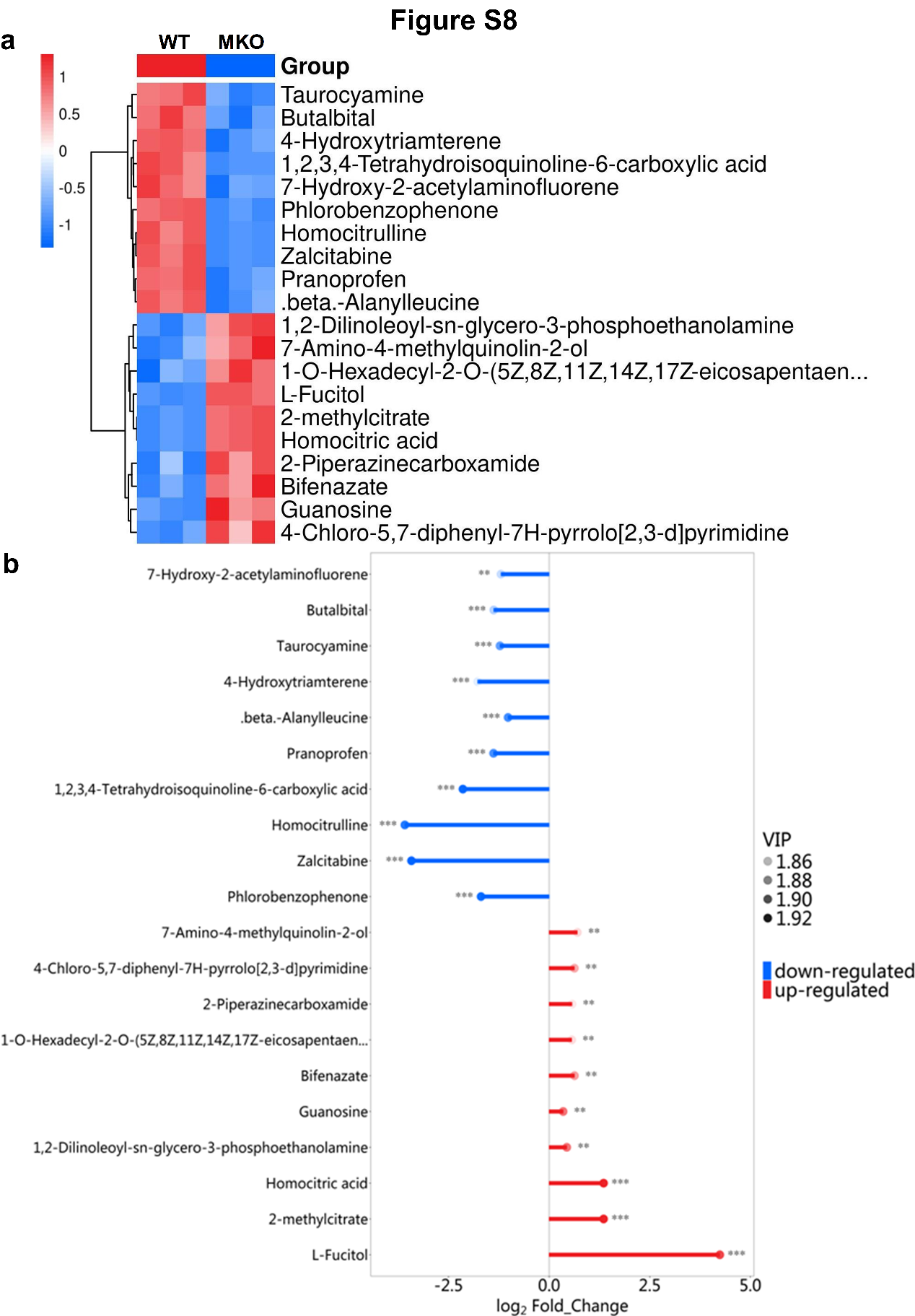


**Figure S8.** Metabolomics analysis for metabolites difference in WT and MKO cardiac tissues under normal conditions. The normal WT and MKO cardiac tissue samples under basal conditions were prepared for metabolomics analysis (n=3). a) Heatmap of metabolomics analysis. The expression abundance of metabolites is evaluated by color change. Color from blue to red means metabolites from low to high abundance. b) Matchstick map of metabolomics analysis. X axis indicates log_2_ Fold Change. The shade of dots indicates VIP value. Blue and red indicate down- and up-regulation respectively. ** p<0.01, *** p<0.001. WT: wild type; MKO: myocardial cell-specific MANF knockout.


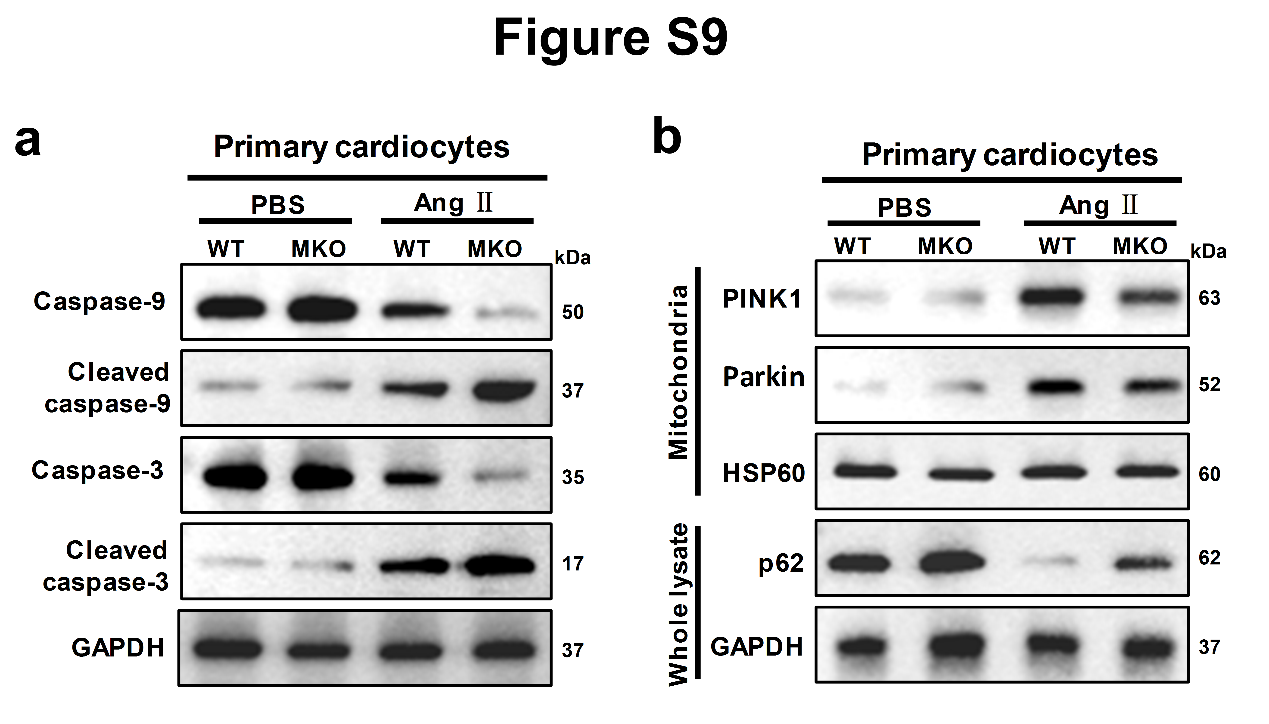


**Figure S9.** MANF deficiency aggravated apoptosis and weakened mitophagy in primary cardiocytes under Ang II stimulation. Primary cardiocytes were extracted from WT and MKO mice, followed by Ang II (0.2 μM) treatment for 48 hours, then WB was performed to detect Caspase-9/3 and Cleaved caspase-9/3 a), as well as PINK1/Parkin in mitochondria and p62 in whole lysate b). Ang II: angiotensin II; PBS: phosphate buffer saline; WT: wild type; MKO: myocardial cell-specific MANF knockout. Data are representative of three independent experiments.


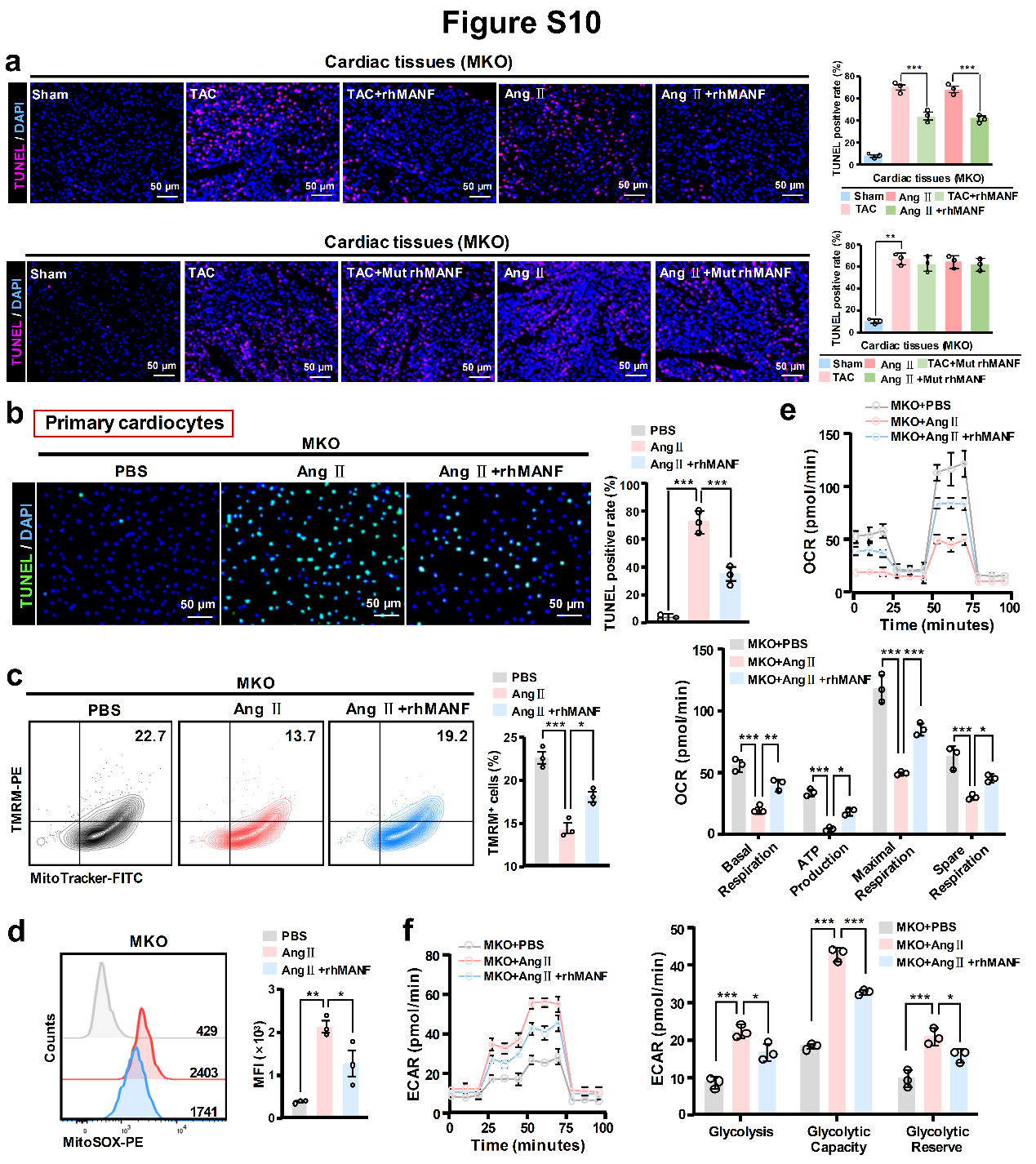


**Figure S10.** Recombinant human MANF protein partly rescued TAC or Ang II-induced cardiomyocyte apoptosis, mitochondrial instability and energy metabolism disturbance in MKO mice. a) TUNEL assay for TAC or Ang II-induced MH cardiac tissues of MKO mice with or without rhMANF or Mut rhMANF treatment respectively (n=3). Scale bar: 50 μm. Primary cardiocytes (1×10^5^ cells/sample) were extracted from MKO mice, followed by Ang II (0.2 μM) treatment with or without rhMANF (20 µg) treatment (n=3). b) TUNEL assay for MKO primary cardiocytes. TUNEL positive rate was calculated. MitoTracker-FITC/TMRM-PE mitochondrial membrane potential detection c) and MitoSOX-PE mitochondrial oxidative stress detection d) were performed. OCR e) and ECAR f) assays were performed to evaluate oxidative phosphorylation and glycolysis respectively. Data are expressed as mean ± SD. * p<0.05, ** p<0.01, *** p<0.001. Sham: sham operation; TAC: transverse aortic constriction; Ang II: angiotensin II; PBS: phosphate buffer saline; MKO: myocardial cell-specific MANF knockout; rhMANF: recombinant human MANF; Mut rhMANF: Mutated recombinant human MANF. Data are representative of three independent experiments.


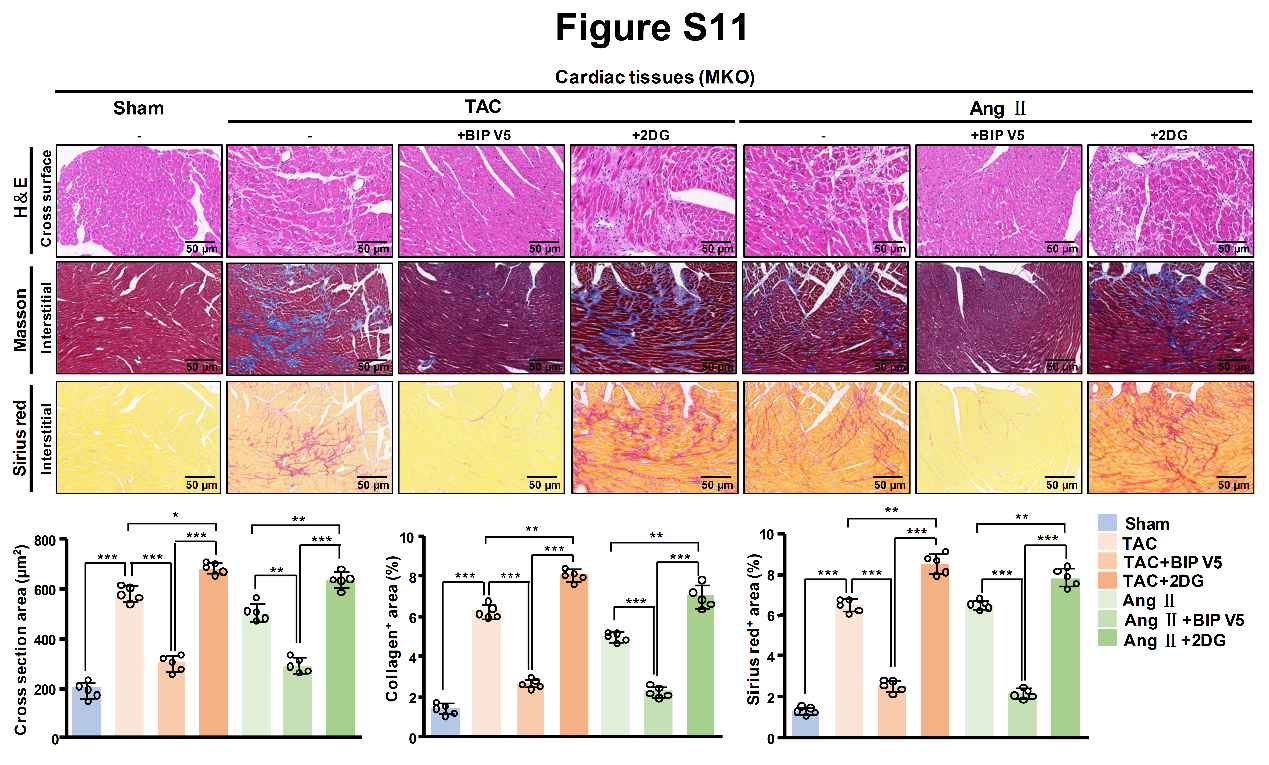


**Figure S11.** BIP V5 and 2DG significantly affected TAC or Ang II-induced MH in MKO mice. MKO mice were used to construct TAC or Ang II-induced MH model, followed by BIP V5 (100 μM) or 2DG (3 mM/kg) treatment (n=5). Cardiac tissues (n=5) were used for HE, Sirius red and Masson staining. Cross section area of HE staining, Sirius red positive area and collagen positive area were calculated. Scale bar: 50 μm. Data are expressed as mean ± SD. * p<0.05, ** p<0.01, *** p<0.001. Sham: sham operation; TAC: transverse aortic constriction; Ang II: angiotensin II; MKO: myocardial cell-specific MANF knockout; BIP V5: BAX Inhibitor Peptide V5; 2DG: 2-Deoxyglucose. Data are representative of three independent experiments.

**Experimental Details**

*Mice:* No inducer was needed for conditional knockout. Male mice were used to eliminate the possible effect of estrogen on experimental results. SPF-class animal laboratory was used for mice breeding (Room temperature: 20°C to 26°C; Daily temperature difference≤4°C; Relative humidity: 40% to 70%; Pressure gradient: 20 to 50 Pa; Lighting: 12 hours alternately; Drinking and eating freely). 9-week-old WT and MKO male mice were used for MH mice model construction and experiments. Random grouping and single-blind manner were carried out in mice grouping, feeding and modeling.

*TAC or Ang II-induced myocardial hypertrophy mice model:* After sacrifice, heart images, cardiac tissues and serum samples were collected. Also, the relevant data was recorded, including mice’s survival rate, heart weight, lung weight and body weight. Cardiac function was monitored by echocardiography to collect and analyze fractional shorting (FS), ejection fraction (EF), left ventricular end-diastolic diameter (LVEDD) and left ventricular end-systolic dimension (LVEDS). For rhMANF protein treatment, rhMANF (15, 30 and 60 μg per mouse) was used for intravenous injection three times per week, which was started at the fourth week after TAC surgery or the second week after Ang II injection. The administration of rhMANF lasted for one week. Twenty-four hours after the last rhMANF injection, all mice were sacrificed. For BIP V5 and 2DG treatment, TAC or Ang II-induced MH MKO mice were administrated by BIP V5 (100 μM, MedChemExpress, NJ, USA, 579492-81-2) and 2DG (3 mM/kg, MedChemExpress, 154-17-6) respectively three times per week.

*Sirius red and Masson staining:* Briefly, Weigert's iron hematoxylin staining, differentiation acidic ethanol solution, Masson solution and ponceau acid fuchs solution treatments were conducted in sequence. After that, weak acid buffer and phosphomolybdic acid solution were used for rinsing, followed by aniline blue staining and ethanol dehydration.

*Western blot (WB):* The involved primary antibodies contain: Anti-MANF (Abcam, Cambridge, UK, ab67271); Anti-GAPDH (Proteintech, Wuhan, China, 60004-1-Ig); Anti-β-ACTIN (Proteintech, 66009-1-Ig); Anti-Cyt-c (Servicebio, Wuhan, China, GB11080-100); Anti-HSP60 (Abcam, ab190828); Anti-BAX (Servicebio, GB114122-100); Anti-Ku70 (Abcam, ab92450); Anti-Caspase-9 (Servicebio, GB115730-100); Anti-Caspase-3 (Servicebio, GB11767C-100); Anti-Cleaved caspase-9 (Cell Signaling Technology, Danvers, USA, #9509); Anti-Cleaved caspase-3 (Cell Signaling Technology, #9664); Anti-p62 (Abcam, ab91526); Anti-Parkin (Abcam, ab77924); Anti-PINK1 (Proteintech, 123274-1-AP); Anti-Complex IV (Abclonal, Wuhan, China, A6564).

*Quantitative real time polymerase chain reaction (qRT-PCR):* Primers in qRT-PCR contain: human MANF, forward 5'-TTTACCAGGACCTCAAAGACAGA

-3' and reverse 5'-TTGCTTCCCGGCAGAACTTTA-3'; mouse MANF, forward 5'-TCTGGGACGATTTTACCAGGA-3' and reverse 5'-CTTGCTTCACGGCAAAAC

TTT-3'; human GAPDH, forward 5'-GGAGCGAGATCCCTCCAAAAT-3' and reverse 5'-GGCTGTTGTCATACTTCTCATGG-3'; mouse GAPDH, forward 5'-AGG

TCGGTGTGAACGGATTTG-3' and reverse 5'-GGGGTCGTTGATGGCAACA-3'; mouse COX6A2, forward 5'-TCCGAACCAAGCCCTTCGC-3' and reverse 5'-GCAA

AGGATTGACGTGGGGA-3'; mouse COX6C, forward 5'- AGGACGTTGGTGTAG

AGGACA-3' and reverse 5'- CCAGAAGACCACGCATCTGT-3'.

*High-throughput proteomic screening:* Firstly, cardiac tissues were used to perform ultrasonic cell lysis for 3 minutes, followed by centrifugation at 12000×rpm for 10 minutes. The supernatant protein solution was collected for BCA protein quantification. 10 μg of each protein sample was used for SDS-PAGE, coomassie blue staining and gel image analysis. The rest protein samples were involved in trypsin enzymolysis and peptide labeling by Tandem Mass Tag (TMT). Ultimately, TMT-labelled protein samples were performed by reversed-phase chromatography separation by Agilent 1100 HPLC and liquid chromatography-mass spectrography (LC-MS) analysis by Thermo EASY-nLC 1000/Thermo Q Exactive HF System. After quality assessment and preprocessing, all collected data was involved in protein expression and function analysis to obtain differentially expressed proteins.

*Single cell RNA sequencing (scRNA-seq):* Microfluidic system and 10×Genomics platform were involved to produce liquid drops containing barcoded gel bead and single cell. Then, the single cell was lysed to connect the intracellular mRNA with barcoded beads for Single Cell GEMs. The reverse transcription was conducted within liquid drops to establish cDNA library, followed by sequencing of single-cell transcriptome. After quality assessment and control of raw data, all collected data was involved in standardized processing, cell heterogeneity, dimensionality reduction, clustering and gene expression analysis.

*RNA sequencing (RNA-seq):* The normal WT and MKO primary cardiocytes were used for RNA-seq to explore gene expression differences in normal myocardial cells without MH. Three WT and MKO mice were divided into WT group (n=3) and MKO group (n=3) respectively, followed by collection of primary cardiocytes. Then, total RNA was extracted for mRNA gathering by Oligo (dT). After mRNA fragmentation, cDNA was synthetized by random primers. PCR amplification was performed to construct cDNA library. After quality assessment, Illumina sequenator was involved for RNA sequencing.

*Metabolomics analysis:* Primary cardiocytes were grinded in the pre-cooling methanol-water solution, followed by ultrasonic cell lysis for 10 minutes. After centrifugation at 12000×rpm for 10 minutes, the supernatant was filtrated to perform LC-MS analysis by Waters ACQUITY UPLC I-Class Plus/Thermo QE HF System. Also, the normal WT and MKO mice (n=3) were used for metabolomics analysis to explore metabolites difference under basal conditions.
